# Engineered platforms for melanogenesis research: Bridging synthetic biology, bioengineering, and biomimetics

**DOI:** 10.1002/btm2.70159

**Published:** 2026-08-21

**Authors:** B. Anika, Arshia Tarkunde, B. S. Swarna, Usha Y. Nayak, Vijendra Prabhu, Raghavendra Rao, A. S. Bharath Prasad

**Affiliations:** ^1^ Department of Public Health Genomics Manipal School of Life Sciences, Manipal Academy of Higher Education Manipal Karnataka India; ^2^ Department of Pharmaceutics Manipal College of Pharmaceutical Sciences, Manipal Academy of Higher Education Manipal Karnataka India; ^3^ Manipal Institute of Technology, Manipal Academy of Higher Education Manipal Karnataka India; ^4^ Department of Dermatology, Venereology and Leprosy Kasturba Medical College, Manipal Academy of Higher Education Manipal Karnataka India

**Keywords:** biomimetic models, cell‐free assays, melanogenesis, pigmentation disorders, synthetic biology

## Abstract

Melanogenesis, the biological process responsible for melanin synthesis, plays a critical role in determining skin pigmentation and providing photoprotection. Dysregulation of this pathway leads to a wide range of pigmentary disorders that affect a significant proportion of the global population. Traditional approaches to studying melanogenesis rely largely on cultured melanocytes and in vivo animal models; however, these systems present several limitations, including concerns related to physiological relevance, ethical constraints, high maintenance costs, and limited scalability. In recent years, synthetic biology has emerged as a powerful framework for engineering controllable biological systems capable of replicating complex cellular pathways with high precision. Although numerous studies have reported individual synthetic biology approaches for pigment production or pathway engineering, the literature lacks a comprehensive synthesis that integrates these strategies within the broader context of melanogenesis research and its translational potential. This review addresses this gap by consolidating advances in synthetic biology platforms used to investigate and manipulate pigmentation biology. We discuss emerging techniques including genetic engineering, heterologous expression systems, biomimetic constructs, and cell‐free assays that enable the reconstruction and modulation of melanin synthesis pathways. These engineered systems allow the development of disease‐specific and patient‐derived pigmentation models, providing new opportunities for mechanistic studies and personalized therapeutic strategies. Conceptually, this review proposes a unified framework that positions synthetic biology as a versatile toolkit for studying melanogenesis while also enabling scalable production of melanin and melanin‐based biomaterials. By bridging developments across molecular engineering, microbial biosystems, and biomimetic technologies, this work highlights how non‐conventional systems can transform both fundamental pigmentation research and translational applications in dermatology and biotechnology.

## Graphical abstract

Synthetic biology and engineered platforms overcome the limitations of conventional melanogenesis models by enabling programmable control of pigmentation biology. These advanced systems provide mechanistic insights and accelerate disease modeling, drug discovery, and translational applications.

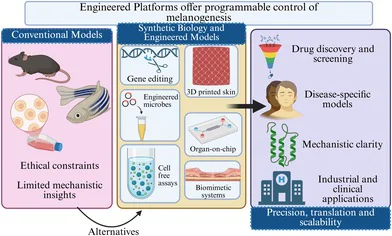

**Translational Impact Statement**

This review lays the foundation for developing next‐generation diagnostic tools and targeted therapies for pigmentation disorders by showcasing synthetic biology and bioengineering platforms that more accurately model human melanogenesis.

## 1 INTRODUCTION

Skin diseases have been documented across human history, from ancient Egyptian papyri and Hippocrates' humoral theory to the classical texts of Ayurveda and Traditional Chinese Medicine.^1^, ^2^, ^3^ For centuries, conditions were treated empirically using plant extracts and observation, as the cellular and molecular drivers of pigmentation remained entirely unknown.^4^, ^5^ Modern dermatological science has since revolutionized our understanding by uncovering these precise molecular mechanisms. We now understand melanogenesis as the complex biological pathway, regulated by both genetic and environmental factors, responsible for synthesizing melanin.^6^ Melanin is a crucial photoprotective pigment that determines skin, hair, and eye color while actively absorbing and dissipating UV radiation to prevent DNA damage.^7^, ^8^ This highly regulated synthesis occurs within melanosomes, which are specialized organelles located inside neural‐crest‐derived melanocytes.^9^ The pathway relies on the conditionally non‐essential amino acid tyrosine, which serves as the primary substrate.^10^, ^11^ The central regulatory enzyme in this entire process is tyrosinase (TYR), a glycoprotein that catalyzes the essential hydroxylation of tyrosine to L‐DOPA and subsequently to L‐Dopaquinone.^12^, ^13^ Understanding this intricate pathway is critical for developing targeted therapies for pigmentary disorders, which were reported by approximately 50% of respondents in a large multinational survey.^1^

Despite the critical need to investigate these pathways, conventional models for studying melanogenesis present profound limitations that hinder translational research. Traditional models primarily consist of 2D in vitro cell cultures, ex vivo explants, and in vivo animal models.^14^ While 2D in vitro cell lines are easily accessible, they do not accurately represent the molecular heterogeneity of human disorders.^15^ Ex vivo human skin explants allow testing in a more physiologically relevant environment but have limitations in capturing molecular heterogeneity.^16^ Furthermore, reliance on in vivo models like mice, zebrafish, and fruit flies presents severe bottlenecks.^17^, ^18^ Beyond the high costs of facility maintenance and growing ethical constraints regarding animal testing, these models exhibit significant species differences.^19^, ^20^, ^21^ Animal models possess divergent protein–protein interactions and distinct genetic backgrounds that fail to accurately represent the vast genetic diversity of human populations. Most critically, these conventional models often fail to capture the deep molecular heterogeneity of human pigmentary disorders, where identical visible phenotypes can emerge from entirely distinct molecular disruptions, leading to inconsistent and unpredictable therapeutic responses.^22^

To circumvent these extensive limitations, researchers are actively adopting synthetic biology as a powerful, customizable alternative. Synthetic biology is an interdisciplinary field that broadly focuses on engineering biological systems to obtain novel or optimized outputs.^23^ Historically, this domain has been closely associated with genetically engineered circuits and programmable gene regulation.^24^ However, as the field has evolved, recent advancements have driven a convergence between conventional synthetic biology, biomaterials design, and biomimetic systems. The engineered pigmentation models discussed in this review encompass: first, classical synthetic biology, which involves direct genetic and metabolic rewiring, encompassing tools like CRISPR‐Cas9, TALENs, and heterologous microbial expression systems; second, bioengineering and tissue engineering, which focuses on the macroscopic fabrication of tissue architecture, including 3D bioprinting and Reconstructed Human Pigmented Skin (RHPS); and third, biomimetic systems, which simulate dynamic functional microenvironments and cellular behaviors (refer to Table 1), including microfluidic skin‐on‐a‐chip platforms, 3D spheroids, co‐cultures, and artificial melanosomes. While these categories represent distinct classical fields, they are increasingly convergent in modern research, as the foundational Design‐Build‐Test‐Learn (DBTL) paradigm is applied throughout their development.^25^ The fundamental rationale for adopting this unified toolkit is its reliance on these shared engineering principles, which allows researchers to create novel, reproducible, and controllable models for pigmentation research (refer to Figure 1). By leveraging these platforms, researchers can overcome conventional bottlenecks in multiple ways:

- Precision Disease Modeling: Genetic engineering tools like CRISPR‐Cas9 allow for the exact insertion or correction of disease‐specific mutations, creating highly accurate genotype‐to‐phenotype models without relying on naturally occurring animal variants.^26^
- Scalability and Speed: Engineered heterologous microbes bypass the slow metabolic kinetics of mammalian cells, enabling the rapid, industrial‐scale biomanufacturing of melanin and targeted pathway dissection.^27^
- Physiological Relevance without Ethical Hurdles: Biomimetic systems replicate the structural complexity of skin.^20^ This allows for highly predictive drug screening while drastically reducing reliance on animal subjects, aligning with ethical Replacement, Reduction, and Refinement (3Rs) principles.
- Absolute Mechanistic Isolation: Cell‐free assays isolate specific reactions from the cellular context, providing controllable, interference‐free environments to precisely study melanogenesis and identify how novel drugs or inhibitors function.^28^

**TABLE 1:** A classification framework outlining the engineered pigmentation models discussed in this review.

Classification domain	Definition & core concept	Specific platforms & technologies	Primary applications in melanogenesis research	References
Classical synthetic biology	Direct genetic, metabolic, and transcriptional rewiring; utilization of programmable gene circuits and biological parts	Targeted Nucleases: CRISPR‐Cas9, TALENs Heterologous Expression: Engineered microbes (e.g., *E. coli*, *Y. lipolytica*). Modular Biosystems: Cell‐free Transcription‐Translation (TXTL)	Precise functional validation of disease mutations; scalable industrial biomanufacturing of melanin; isolated enzymatic pathway dissection	26, 27, 29, 30
Bioengineering & tissue engineering	Macroscopic fabrication of tissue architecture and structural reconstruction of the epidermal‐melanin unit	Tissue Fabrication: 3D Bioprinting using custom cellular bioinks Standardized Constructs: Reconstructed Human Pigmented Skin (RHPS) & Epidermis (RHPE) Commercial Models: MelanoDerm™, EpiSkin™	Creation of standardized, reproducible models for high‐throughput preclinical compound screening; development of autologous grafts for regenerative medicine	31, 32, 33, 34
Biomimetic systems	Simulation of dynamic functional microenvironments, physiological intercellular signaling, and sub‐cellular behaviors	Dynamic Culture: Microfluidic skin‐on‐a‐chip 3D Cellular Assembly: Melanocyte spheroids Cellular Interaction: Primary melanocyte‐keratinocyte co‐cultures Synthetic Organelles: Artificial/polymeric melanosomes	Predictive toxicity testing; long‐term exposure studies mimicking systemic/hormonal triggers (e.g., pregnancy‐induced melasma); localized and photo‐triggered UV protection	35, 36, 37, 38, 39

*Note*: Classical Synthetic Biology involves direct genetic and metabolic manipulation, whereas Bioengineering and Biomimetic Systems emphasize the macroscopic replication of tissue architecture and the simulation of dynamic physiological environments, respectively. These distinct fields converge through the shared application of Design‐Build‐Test‐Learn (DBTL) engineering principles, forming a unified toolkit for advanced pigmentation research.

**FIGURE 1:**
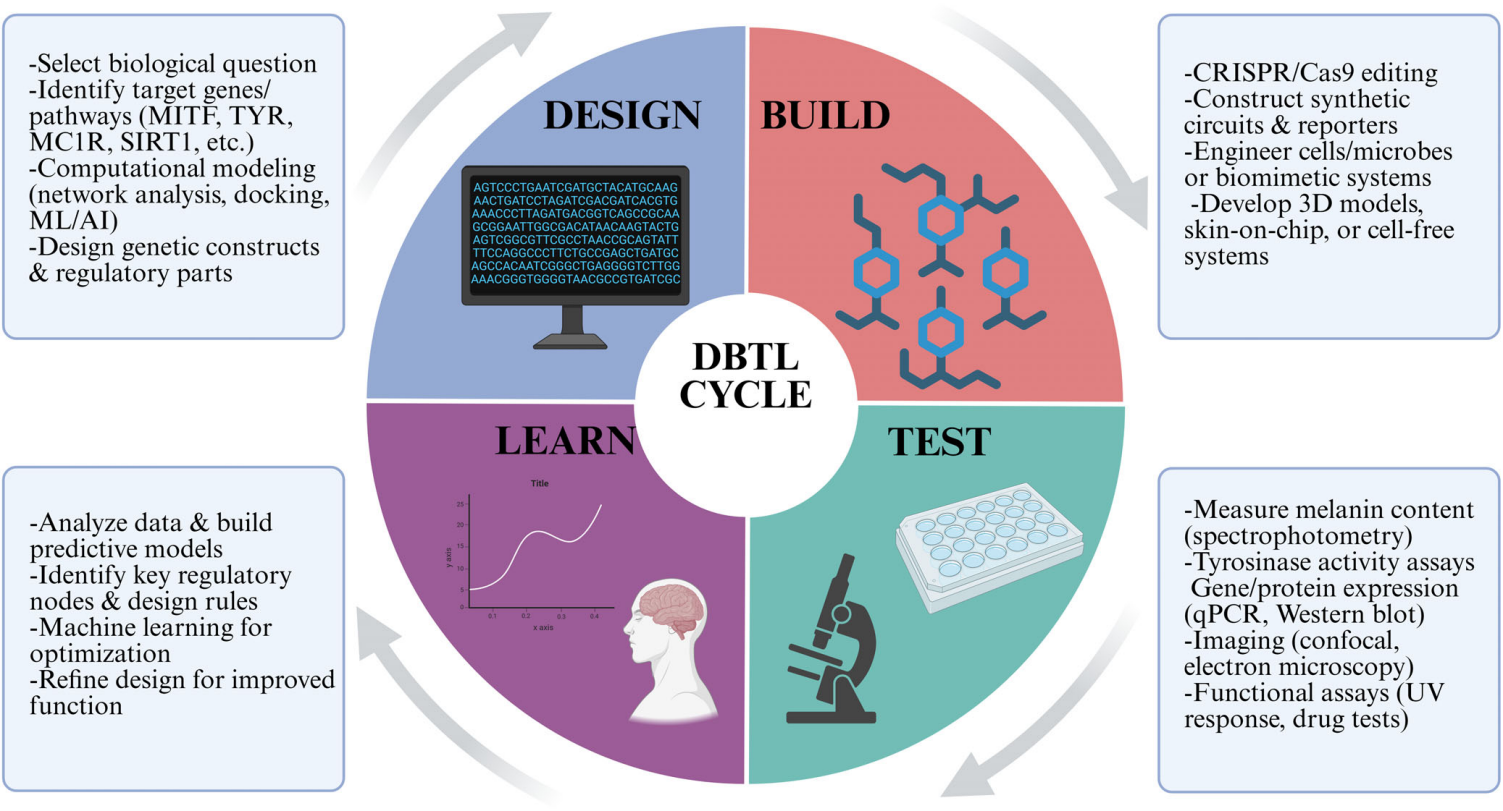
The Design‐Build‐Test‐Learn (DBTL) cycle applied to melanogenesis. The “Design” phase identifies biological targets and pathways computationally. The “Build” phase constructs the physical models, utilizing CRISPR‐Cas9, engineered microbes, or 3D biomimetic skin. The “Test” phase systematically evaluates these platforms through functional assays and melanin quantification. Finally, the “Learn” phase uses data analysis to refine the initial design, creating a continuous feedback loop for system optimization.

Ultimately, by replacing outdated, unpredictable, and ethically constrained conventional models with highly controllable engineered biosystems, this comprehensive framework represents an advancement in melanogenesis research, enabling deeper insights and optimized therapeutic strategies to treat pigmentary disorders.

## 2 CONVENTIONAL PIGMENTED MODELS IN MELANOGENESIS RESEARCH

Understanding the biological mechanisms of melanogenesis has long relied on the use of model organisms and in vitro models that naturally exhibit pigmentation. These models possess endogenous pigment cells, which allow direct observation of melanosome behavior and dynamic responses to environmental and pharmacological stimuli.^40^ Human skin is a stratified organ, composed primarily of epidermis, dermis, and hypodermis.^41^ Melanocytes, the melanin‐producing cells, are primarily located in the basal layer of the epidermis alongside keratinocytes, sensory receptors, and resident immune cells.^42^ Inside melanocytes, the production of melanin occurs in an organelle called the melanosome, which is in the perinuclear region of the cell. Upon maturity, the melanosomes are transported via microtubules to the cell periphery for transfer to neighboring keratinocytes in the skin, contributing to skin pigmentation and photoprotection.^43^ Pigmentation is therefore governed by both melanocyte‐intrinsic processes and intercellular interactions within the epidermal microenvironment.

### 2.1 Cell lines

B16F10 mouse melanoma cell lines are widely used for studying melanogenesis‐related processes owing to their high melanin‐producing capacity. It is often considered the standard model for melanogenesis and pigmentation research. This cell line is a subclone of the original B16 cell line and produces black melanin pigment, making it ideal for visualizing pigmentation.^44^ They are also responsive to external stimuli such as α‐MSH and UV radiation, making them ideal for pharmacological screening.^45^, ^46^

Human melanoma cell lines, such as the Sk‐Mel series (Sk‐Mel‐1, Sk‐Mel‐3, Sk‐Mel‐5, Sk‐Mel‐28, Sk‐Mel‐30, Sk‐Mel‐147), are commonly used to evaluate various components of the melanogenesis pathway, including melanin content, tyrosinase activity, and MITF regulation.^47^, ^48^, ^49^, ^50^ The A375 cell line is frequently used to study apoptosis, drug response, and signaling pathways such as MAPK/ERK.^51^ The MNT‐1 human melanoma cell line is highly pigmented and widely used to investigate melanosome biogenesis, trafficking, and melanin synthesis. These cells express high levels of tyrosinase and dopachrome tautomerase (DCT), and produce eumelanin, making them particularly valuable for imaging and biochemical analyses.^52^

Primary Melanocytes, such as HeMa cells, are directly isolated from skin biopsies and exhibit characteristics closer to in vivo conditions. Unlike immortalized cell lines, primary cells only survive for a limited number of passages after which they attain senescence.^53^ These cells enable personalized studies on pigmentation, especially when derived from donors of diverse ethnic backgrounds.

Human keratinocytes (HaCaT), a component of the skin epidermis along with melanocytes, are also used in melanogenesis research even though they are non‐pigmented cells. They are majorly used in co‐culture systems to replicate epidermal architecture and investigate the transfer of melanin from melanocytes to keratinocytes. Other supporting cell types, such as dermal fibroblasts, can also be integrated into tri‐culture skin models to better replicate in vivo microenvironments.^15^ The different cell lines are compared in Table 2.

**TABLE 2:** Cell line models used in melanogenesis research and pigmentation studies.

Sl. no.	Type of cell line	Advantage(s)	Disadvantage(s)	Example(s)	Citation(s)
1.	Mouse Melanoma Cell Lines	Mouse cells can be reimplanted into mice to study the progression of the disorder, in case of non‐availability of previously established animal models.	Mouse genes may not be identical to human genes. Protein–protein interactions differ in mice and humans. Cell lines derived from mice do not represent the vast genetic diversity of humans.	B16F10 Cell line	44
2.	Human Melanoma Cell Lines	These cells enable the study of melanin synthesis in a controlled and reproducible environment.	These cell lines do not accurately represent the molecular heterogeneity of human disorders.	Sk‐Mel Series, MNT‐1, A375 Cell line	49, 52
3.	Primary Melanocytes	Primary cell lines enable personalized studies on pigmentation. They provide conditions that mimic the in vivo conditions better.	These cells survive only for a limited number of passages before reaching senescence.	HeMa Cell line	53
4.	Keratinocytes	Keratinocytes are used in co‐culture models to mimic epidermal architecture and to describe the transfer of melanin from melanocytes to keratinocytes. This creates a more physiologically relevant model.	Keratinocytes do not produce melanin.	HaCaT Cell line	15

### 2.2 In vivo models

Mouse models (*Mus musculus*) are a popular tool used by researchers to delve deeper into pigmentary disorders and melanogenesis, due to the high degree of genetic similarity and visible pigmentation phenotypes.^54^ Melanin‐related phenotypes in mice can be modulated via genetic modifications (e.g., MC1R, TYR, or OCA2 knockouts), UV exposure, or pharmacological treatments.^55^ C57BL/6 mice are considered the gold standard for hyperpigmentation studies due to their naturally dark coat. These mice are also responsive to UV light, making the induction of hyperpigmentation easier for subsequent studies.^18^ Physical methods such as UV induction and epilation are typically used to induce pigmentation in mice.^55^, ^56^ Additionally, chemical treatment with haptens and other signaling molecules, or genetic modification in loci specific to pigmentation modulation can also be used to promote melanin synthesis.^57^

Zebrafish, also known as *Danio rerio*, are an alternative to mouse models. The transparent body of the zebrafish embryos makes them an attractive option to visualize melanin development in real time. Zebrafish are small, inexpensive, and reproduce rapidly, making them ideal model organisms. Additionally, the origin of melanocytes from neural crest cells in zebrafish indicates a high degree of homology between humans and zebrafish. The genetic tractability and optical clarity of zebrafish embryos allow high‐throughput screening of melanogenic or depigmenting agents in vivo.^58^, ^59^ Similar to mice and humans, melanin synthesis can be induced or inhibited by chemical compounds such as 5‐Hydroxytryptamine and Phenylthiourea, respectively.^60^, ^61^

The African clawed frog (*Xenopus laevis*) produces embryos that possess melanophores derived from neural crest cells, thus making it an ideal system to study the spatiotemporal regulation of melanogenesis along with the developmental and genetic factors.^62^ Xenopus embryos exhibit melanin deposition at the animal pole that is tyrosinase‐dependent but MITF‐independent, which offers a unique model to study the non‐canonical pathways of melanogenesis. Furthermore, their large embryonic size facilitates microsurgical manipulations and in situ hybridization for spatial gene expression studies during melanogenesis.^63^ Studies have induced pigmentation in this model through treatment with α‐Melanocyte Stimulating Hormone, placing the larvae on a dark background to trigger background adaptation and genetic manipulation of genes in the MITF family.^64^, ^65^

In addition to the mentioned models, guinea pigs, swine models, chick embryos, and *Drosophila melanogaster* are also typically used to help in a broader understanding of melanogenesis.^66^, ^67^, ^68^, ^69^

These conventional models remain indispensable in melanogenesis research, offering varied molecular and organismal insights. However, their limitations have prompted growing interest in synthetic and modular systems that provide enhanced control, reproducibility, and ethical flexibility.

A comparative analysis of the aforementioned in vivo models is presented in Table 3.

**TABLE 3:** In vivo models used in melanogenesis research and pigmentation studies.

Sl. no.	Type of in vivo model	Advantage(s)	Disadvantage(s)	Citation(s)
1.	Mouse model (*Mus musculus*)	Mice have high degree of genetic similarity to humans. Models which have visible pigmentation phenotypes are available.	Ethical concerns High costs for maintenance and upkeep of the animals.	55, 70
2.	Zebrafish (*Danio rerio*)	Zebrafish embryos have a transparent body which allows for real‐time and visual analysis of pigmentation. Melanocytes in zebrafish originate from neural crest cells, indicating a high degree of homology with humans. These are small and reproduce rapidly.	High cost for zebrafish facility maintenance. Ethical concerns	58
3.	African Clawed Frog (*Xenopus laevis*)	Embryos of this model offer a unique model to study the non‐canonical pathways of melanogenesis. Melanophores are derived from neural crest cells, indicating similar descent to the melanocytes of humans.	Ethical concerns	63
4.	Guinea Pig	The skin of guinea pigs undergoes UV induced hyperpigmentation, making it useful in studying the pathways involved in such processes.	It is a less prevalent model to study melanogenesis, and hence, not a lot of data is available regarding the same.	67
5.	Swine	Swine skin shows high degree of similarity to human skin, and can be used to study pigmentation disorders, as well as check the permeability of drugs and compounds used to treat the disorders.	High cost of maintenance. Ethical concerns.	66
6.	Chick Embryo	This model facilitates live, in vivo observation of the development of melanophores in the body, which is valuable for studying the teratogens or drugs that affect pigmentation during the developmental phase.	Ethical concerns This model lacks the physiological complexity of a mammal.	9, 69
7.	Fruit Fly (*Drosophila melanogaster*)	Fruit fly has a high reproductive rate, and a short life cycle. It has a small and well catalogued genome, making it ideal for genetic studies. They are inexpensive to maintain, and do not require a lot of space.	Lacks the physiological complexity of mammals. Flies are evolutionarily farther away from humans than the other model organisms.	68

## 3 GENETIC ENGINEERING APPROACHES

Since the establishment of the Central Dogma of Molecular Biology, continuous efforts have been made toward developing various methods to understand and manipulate DNA, the Blueprint of Life.^71^ The advent of the post‐genomic era brought with it a greater understanding of the genes essential for each life process. It thus expedited the advancement of these methods that genetically engineer the cell to bring about the desirable changes.^72^ Modifications done to specific biological pathways in an organism, including knockouts, knock‐ins, and replacements, are facilitated by the ability of modern methods to target specific locations in the genome.^71^ In the context of melanogenesis, altering the transcription, translation, and expression of genes and proteins involved in the melanin synthesis pathway can result in optimized systems that enable further research in the area.

### 3.1 CRISPR‐Cas9

CRISPR‐Cas9 (Clustered Regularly Interspaced Short Palindromic Repeats‐CRISPR associated protein 9) is a genome editing tool adapted from the bacterial immune system.^26^ The Cas9 enzyme is an endonuclease that is aided by a guide‐RNA and specific spacers to precisely cut DNA at a targeted location. Once the cut is made, the DNA repair mechanisms, namely, the Non‐Homologous End Joining or the Homology Directed Repair, are harnessed to disable or insert a new gene sequence, thus facilitating gene modifications.^73^ This mechanism is schematically represented in Figure 2.

**FIGURE 2:**
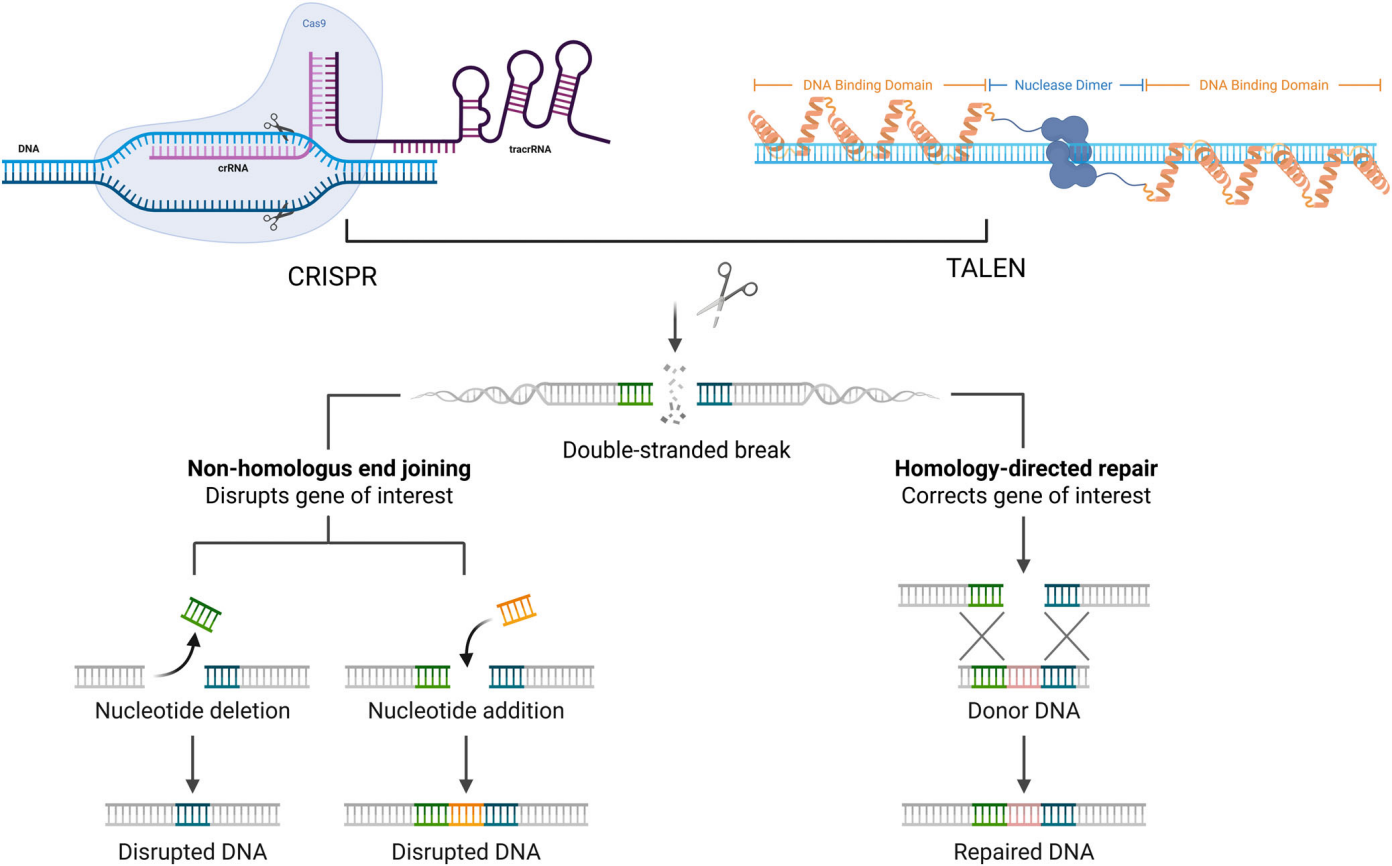
Schematic illustration of genome editing nucleases inducing double‐strand break (DSB) in target DNA. DSBs activate the DNA repair pathways: The error‐prone nonhomologous end joining (NHEJ) or accurate but template dependent homologous recombination (HR). Adapted from,^74^ under CC BY 4.0.

The CRISPR‐Cas9 system enables the precise engineering of in vitro models to study bidirectional pigmentary dysfunctions by targeting specific regulatory genes, such as sirtuins. For instance, knocking out the *SIRT1* gene in B16F1 murine melanoma cells effectively downregulates melanogenesis. *SIRT1* is a sirtuin associated with Histone Deacetylases (HDACs), which manages essential processes such as gluconeogenesis and stress responses in the nucleus.^75^ The loss of functional *SIRT1* alters the p38 and ERK signaling pathways, leading to a significant reduction in MITF expression and subsequent melanin synthesis, thereby providing a highly controlled model for studying hypopigmentation disorders.^76^ Conversely, applying the same CRISPR‐Cas9 methodology to knock out the *SIRT7* gene yields the opposite phenotype. *SIRT7* ablation significantly upregulates melanin synthesis by increasing the expression of MITF and its downstream melanogenic enzymes, including tyrosinase, TRP‐1, and TRP‐2.^77^

The *SIRT1* knockout model can be used to study oxidative‐stress‐driven disorders like vitiligo, while the *SIRT7* knockout model aligns with features of melasma and lentigines, making them valuable tools to dissect the molecular underpinnings of hypo‐ and hyperpigmentation, respectively.

Oculocutaneous albinism (OCA) represents a group of autosomal recessive disorders characterized by severe hypopigmentation, often driven by specific genetic alterations such as the T373K missense mutation that abolishes tyrosinase activity. Synthetic biology platforms like CRISPR‐Cas9 enable the functional validation and targeted correction of these specific genetic variants to create precise disease models. For example, researchers successfully utilized the CRISPR‐Cas9 system to correct the T373K mutation in an in vivo albino model, replacing the mutant allele with the wild‐type sequence. This targeted intervention effectively restored tyrosinase function and produced a marked increase in melanin content.^78^ By successfully reverting a specific single‐nucleotide polymorphism, this approach establishes a highly accurate genotype‐to‐phenotype model that closely mimics the human condition. Ultimately, such models extend beyond basic disease simulation by providing a critical platform for studying the long‐term clinical effects and heritability of genetic interventions, serving as a vital proof‐of‐concept for somatic gene therapy in congenital pigmentary disorders.

While CRISPR‐Cas9 presents immense potential for correcting mutations in pigmentary disorders, its clinical and experimental translation requires a critical evaluation of its limitations, particularly regarding off‐target effects, immune stimulation, and long‐term stability.^79^, ^80^ Targeting regulatory genes like *SIRT1* or *SIRT7* yields valuable mechanistic models, but unintended off‐target cleavage, often caused by mismatches between the sgRNA and non‐target DNA, could disrupt essential non‐targeted regulatory networks, leading to unpredictable cellular toxicity or oncogene activation.^79^ To mitigate these off‐target effects, researchers are increasingly optimizing sgRNA sequences and utilizing high‐fidelity Cas9 variants or modified Cas9 nickases to enhance precision.^79^ Furthermore, achieving long‐term stability of the edited phenotype in live models remains challenging. Because human skin is a continuously renewing tissue, the relatively low delivery efficiency, restricted cargo capacity, and potential immunogenicity of standard viral vectors can limit the durable persistence of the genetic correction across cell divisions, necessitating the development of more stable, localized non‐viral delivery vehicles.^79^, ^81^

Recent advancements in bioengineering have introduced non‐viral, localized delivery systems that successfully overcome the skin barrier. For instance, Li et al. demonstrated the successful transdermal delivery of a CRISPR/Cas9 ribonucleoprotein (RNP) targeting melanocytes using a polyamines‐modified thermosensitive hydrogel. This innovative vehicle remains fluid at 4°C for easy injection but forms a stable gel at body temperature (37°C), allowing for the sustained, localized release of gene‐editing components. The hydrogels are engineered with a positive surface charge, helping it bind effectively to the negatively charged surface of the tissues and enhancing the cellular uptake. In vivo studies conducted on this model showed no systemic toxicity, while achieving 46% gene mutation frequency. This makes the hydrogel model a highly translatable framework for future research.^81^ In addition to localized delivery systems, ex vivo genome editing strategies involving correction of patient‐derived skin cells followed by autologous transplantation have emerged as promising therapeutic approaches for genetic skin disorders. In this strategy, cells isolated from a patient biopsy are genetically corrected in vitro and expanded as epidermal sheets before grafting back to the patient, allowing both safety validation and durable gene expression.^82^, ^83^ Such genome editing strategies can facilitate targeted modulation of melanogenesis pathways in acquired disorders including vitiligo and hyperpigmentation conditions such as melasma.

### 3.2 TALENs

Transcription Activator‐Like Effector Nucleases (TALENs) are composed of a DNA‐binding domain and a FokI nuclease domain. Two TALENs bind opposite strands of DNA, allowing the FokI nuclease domains to inflict a double‐stranded break. The break is then repaired through non‐homologous end joining or through the homology‐directed repair, thus enabling targeted gene editing.^84^ Although there are limited studies exploring the role of TALENs for editing melanogenesis‐related genes, their high specificity and ability to induce breaks in the DNA strands indicate their potential in the analysis of pigmentation pathways.^85^ Figure 2 schematically represents this mechanism.

In a study about *Euthynnus affinis* (a species of tuna fish), researchers used TALENS to disrupt the slc24a5 gene. This gene disruption led to reduced melanin expression in the retinal pigment epithelium during the early development and resulted in hypopigmented patches on the body.^29^ Another study on Zebrafish showed the efficacy of the GoldyTALEN modified scaffold in inducing locus‐specific DNA breaks in somatic and germline tissues.^86^

Genetic engineering offers precision, reproducibility, and control that traditional methods cannot. This control enables the creation of disease‐specific models, industrial‐scale melanin production systems, or the simulation of pigmentary conditions without relying solely on naturally occurring variants.

## 4 HETEROLOGOUS EXPRESSION SYSTEMS

The expression of genes from one species into another species or system, which does not typically produce the protein coded by the gene, is termed heterologous gene expression systems. This shows promise in research by aiding the study of melanogenesis in organisms that are better suited to the lab environment or have faster growth and metabolism rates. These techniques bypass the need for naturally melanogenic organisms by allowing melanin to be expressed in more convenient, rapidly dividing microbial hosts.^87^

The translational viability of a microbial production system is largely dictated by its kinetic growth and biomass‐specific substrate uptake rates. *Vibrio natriegens*, a non‐pathogenic marine bacterium, represents an optimal host due to its exceptionally rapid doubling time of approximately 10 min. By engineering *V. natriegens* to heterologously express a prokaryotic tyrosinase gene (*tyr1* from *Bacillus megaterium*) through cloning using a plasmid, researchers have successfully bypassed the slow metabolic kinetics of traditional models. When supplemented with necessary precursors such as L‐tyrosine and copper, this engineered microbial platform can generate visible melanin within just 15 min, achieving complete saturation in the culture medium within 2 h.^27^ This drastically accelerated production rate underscores the immense practical value of synthetic biology in establishing highly scalable, high‐throughput biomanufacturing platforms for melanin and its derivatives. In the same study, optogenetic control was experimentally demonstrated by placing tyrosinase expression under a light‐responsive transcriptional repression system, enabling blue‐light‐mediated derepression of the promoter and rapid induction of melanin synthesis. Compared to IPTG‐based chemical induction, optogenetic regulation provided reversible, spatially precise, and temporally tunable control over tyrosinase expression without the need for exogenous inducers. Characterization and analysis revealed the melanin produced by *V. natriegens* to be consistent with the DHICA derived melanin (one of the main intermediates in the synthesis of eumelanin).^27^

While some bacterial species produce melanin naturally, their pigment yields are often highly inconsistent under standard laboratory conditions.^88^ To overcome this production bottleneck, researchers have successfully transferred specific melanin biosynthesis genes into highly tractable, easily engineered hosts like *Escherichia coli*. For instance, the heterologous expression of diverse tyrosinase genes, such as *melA* from *Rhizobium etli* or *mel* from *Streptococcus antibioticus*, enables controlled and efficient melanin production when the system is supplied with appropriate precursors.^89^ Similarly, engineering *E. coli* to express the *hppd* gene (encoding 4‐hydroxyphenylpyruvate dioxygenase) derived from species like *Pseudomonas aeruginosa* or *Streptomyces fungicidicus* allows for the targeted, high‐yield biosynthesis of pyomelanin via the homogentisic acid pathway.^90^, ^91^

Beyond simply scaling up natural pigments, these heterologous microbial platforms can be designed as modular systems to synthesize structurally diverse, non‐natural melanin variants. By co‐expressing multiple biosynthetic enzymes, such as tyrosinase, tyrosinase ammonia lyase, and lysine decarboxylase, bioengineers can direct metabolic flux to produce eumelanin, allomelanin, or specialized melanin‐diamine complexes. Supplementing these engineered systems with specific precursors like caffeic acid and C‐5 diamines not only significantly accelerates production rates but also yields modified melanin complexes specifically optimized for industrial applications, such as fabric dyeing.^92^

In addition to bacterial hosts, eukaryotic microbes, particularly yeasts, serve as highly valuable platforms for both studying endogenous melanogenesis and biomanufacturing pigment. Fungal species such as *Candida albicans*, *Exophiala dermatitidis*, and *Aureobasidium pullulans* synthesize native melanins that can be enzymatically extracted to study the pigment's structural and protective roles during infection or environmental stress.^93^, ^94^, ^95^, ^96^ From a translational perspective, non‐pathogenic yeasts like *Yarrowia lipolytica* offer an excellent system for generating commercial‐grade biomaterials. When supplied with exogenous L‐tyrosine, *Y. lipolytica* secretes pyomelanin that exhibits potent photoprotective and antioxidant properties, demonstrating significant promise for integration into dermatological and cosmetic formulations.^94^, ^97^ The diverse production of melanin across these fungal species is illustrated in Figure 3. This figure outlines the scalable heterologous production of melanin by engineered microorganisms, primarily focusing on fungal hosts. It also highlights the diverse commercial, cosmetic, and pharmaceutical applications for this microbially synthesized pigment.

**FIGURE 3:**
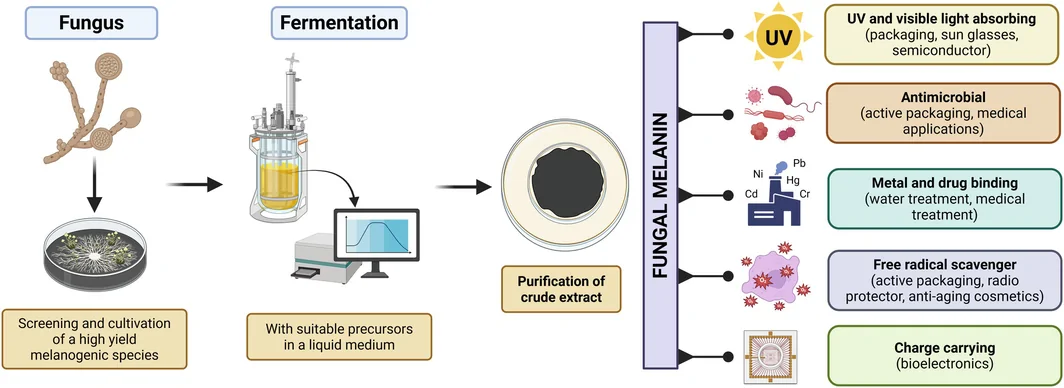
Production of melanin by micro‐organisms (mainly fungi) and their applications. Adapted from^88^ under CCBY 4.0.

These heterologous platforms not only enable large‐scale pigment production but also allow a systematic study of melanin biosynthesis steps under controlled conditions. Researchers can express individual or multiple enzymes involved in melanin synthesis (such as tyrosinase, DOPA oxidase, and dopachrome tautomerase) to study their regulation, activity, and interaction.^98^, ^99^ Production of recombinant melanin pigment is on the rise due to the vastly varying melanin production titers in the naturally melanin‐synthesizing bacteria and fungi. Often, melanin pigment is used in cosmetics, pharmaceuticals, fabric dyeing, and research‐based activities. Melanin pigment can be extracted from certain biological or synthetic systems for use in these sectors.^100^

Heterologous systems engineered via synthetic biology utilize robust, rapidly dividing microbial hosts. For instance, *Vibrio natriegens* boasts a doubling time of roughly 10 min, facilitating the saturation of melanin in culture medium within just 2 h, a kinetic rate drastically superior to mammalian cell lines.^27^ Despite the remarkable scalability and efficiency of microbial systems for industrial melanin production, a critical limitation is their lack of direct physiological relevance to human melanocyte biology. Engineered prokaryotic hosts like *E. coli* or *V. natriegens* lack the complex intracellular compartmentalization (melanosomes) and eukaryotic post‐translational modification machinery (PTMs) required to accurately process native mammalian tyrosinase.^27^, ^91^ Furthermore, single‐cell microbes cannot replicate the crucial intercellular signaling and structural dynamics of the human epidermal‐melanin unit. Consequently, while these platforms possess high translational feasibility for the scalable biomanufacturing of melanin biomaterials and isolated enzymatic screening,^87^ they possess a significant translational gap when attempting to model the complex, multicellular pathophysiology of human pigmentary disorders.

Synthetic biology addresses this limitation through two primary strategies: the bioprospecting of prokaryotic enzyme analogues and the optimization of eukaryotic microbial hosts. To bypass the need for eukaryotic PTMs, researchers frequently engineer bacteria to express prokaryotic tyrosinase homologues, such as tyr1 from *Bacillus megaterium* or hppd from *Streptomyces fungicidicus*, which fold and function independently of the ER/Golgi.^27^, ^91^ Alternatively, when the study of highly complex, heavily modified eukaryotic enzymes is required, researchers utilize yeast species such as *Yarrowia lipolytica*. As eukaryotic hosts, engineered yeasts retain the intracellular machinery necessary for proper protein glycosylation, bridging the gap between scalability and enzymatic complexity.^87^

The use of engineered microorganisms for melanin production and melanogenesis studies also raises biosafety considerations. Although most reported systems rely on well‐characterized laboratory strains and are confined to controlled research or industrial settings, risks associated with unintended environmental release or horizontal gene transfer must be considered. These concerns are mitigated through established biosafety practices, including physical containment, the use of auxotrophic or non‐pathogenic host strains, and, where appropriate, genetic safeguards that limit survival outside defined conditions. When implemented within existing biosafety and regulatory frameworks, engineered microbial platforms offer a controlled and scalable alternative to animal‐based or chemically intensive approaches, while maintaining an acceptable biosafety profile.

## 5 BIOMIMETIC SYSTEMS

In recent years, the use of biomimetic systems—engineered models that mimic biological processes—has gained popularity. From prosthetic limbs with biomimetic joints to 3D printed tissues, these biomimetic systems have been prominently utilized in the medical space to emulate biological functions. They have found prominent roles in melanogenesis research, where models emulating specific disorders of the skin aid in a better understanding of the internal processes.

Transitioning from basic monocultures to biomimetic co‐culture systems significantly enhances the physiological accuracy of in vitro skin models. For instance, co‐culturing primary melanocytes and keratinocytes isolated directly from the skin of vitiligo patients creates a highly relevant, patient‐specific disease platform. Unlike standard models, these primary co‐cultures preserve the native pathophysiology of the condition; when subjected to pharmacological screening, vitiligo‐derived co‐cultures exhibit distinctly altered drug responses, showing notable resistance to melanogenic stimulators and heightened sensitivity to inhibitors compared to healthy control cultures.^37^ By accurately recreating the complex microenvironment of diseased skin, these co‐cultures provide a vastly superior and highly predictive preclinical proxy for screening targeted therapies. Representative images of these co‐cultured networks are depicted in Figure 4. This figure displays representative images of biomimetic co‐cultures containing primary melanocytes and keratinocytes from healthy controls versus *Vitiligo vulgaris* patients. These models recreate native epidermal interactions to accurately assess pharmacological responses to specific melanogenic modulators.

**FIGURE 4:**
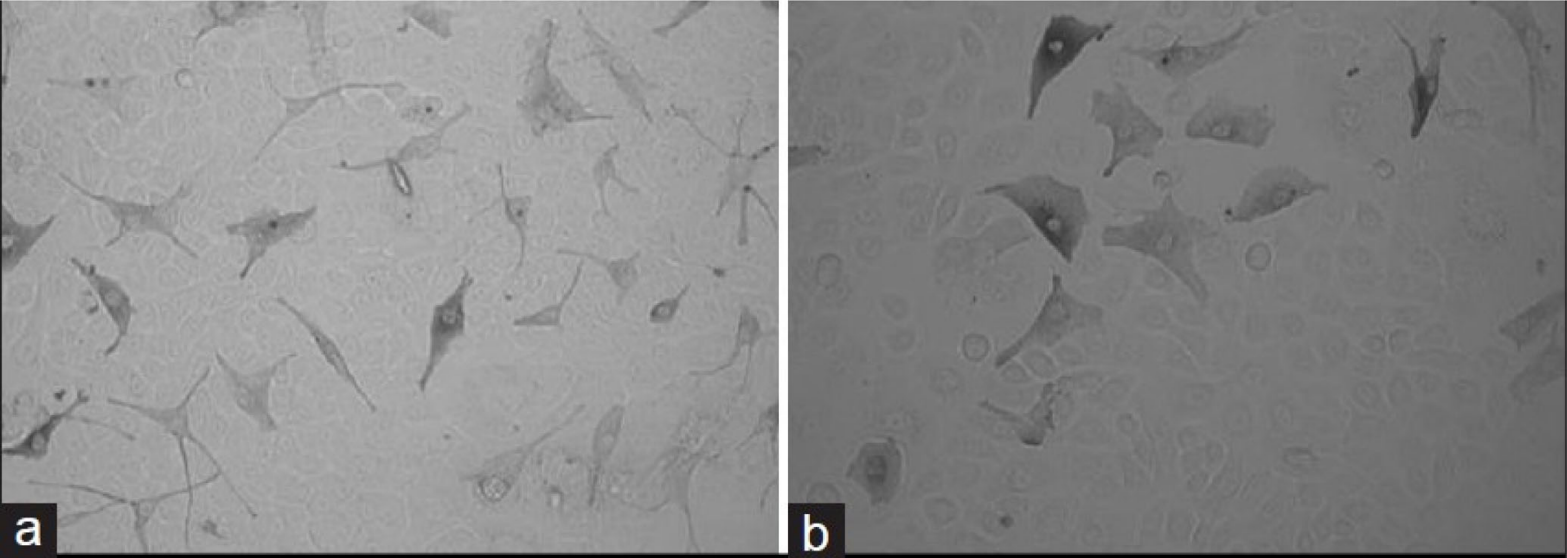
Representative pictures of the cocultured primary melanocytes and keratinocytes from control (a) and Vitiligo vulgaris patients (b). Reproduced under Open Access charter from: Kumar et al.^37^

Building upon the benefits of co‐culture, advancing to three‐dimensional (3D) spheroid models addresses the inherent limitations of flat 2D monolayers, where isolated melanocytes notoriously lose their melanin‐producing capabilities. By cultivating melanocytes on non‐adhesive substrates to promote self‐assembly into 3D spheroids, researchers can maintain the cells' functional activity and significantly amplify melanin synthesis.^36^ These 3D architectures provide a highly sensitive and physiologically accurate platform for pharmacological screening. Studies suggest that when treated with the skin‐lightening agent fucoxanthin, 3D melanocyte spheroids demonstrated a more pronounced and rapid downregulation of melanogenic genes and melanin synthesis than established commercial tissue equivalents like MelanoDerm™.^36^, ^101^ Consequently, 3D spheroids serve as an optimized, high‐throughput testing ground for discovering and validating novel depigmenting compounds.

To capture even greater physiological complexity, bioengineers are integrating these multi‐cellular models into dynamic skin‐on‐a‐chip platforms capable of simulating systemic influences, such as continuous blood flow and hormonal fluctuations. A microfluidic device incorporating a bilayer hydrogel, comprising a dermal layer of fibroblasts and an epidermal layer of melanocytes, has been developed to specifically model chloasma, a pregnancy‐induced form of melasma. By precisely regulating oxygen gradients and circulating 17β‐estradiol levels through the chip's fluidic channels to mimic the physiological state of pregnancy, this biomimetic platform successfully reproduces the exact hyperpigmentation patterns observed clinically in pregnant women.^35^ Such advanced, perfused 3D culture systems demonstrate how bioengineering can be leveraged to study complex, hormone‐driven pigmentary disorders, offering a robust preclinical pipeline for targeted dermatological therapies. Figure 5 shows a representative image of a skin‐on‐a‐chip platform.

**FIGURE 5:**
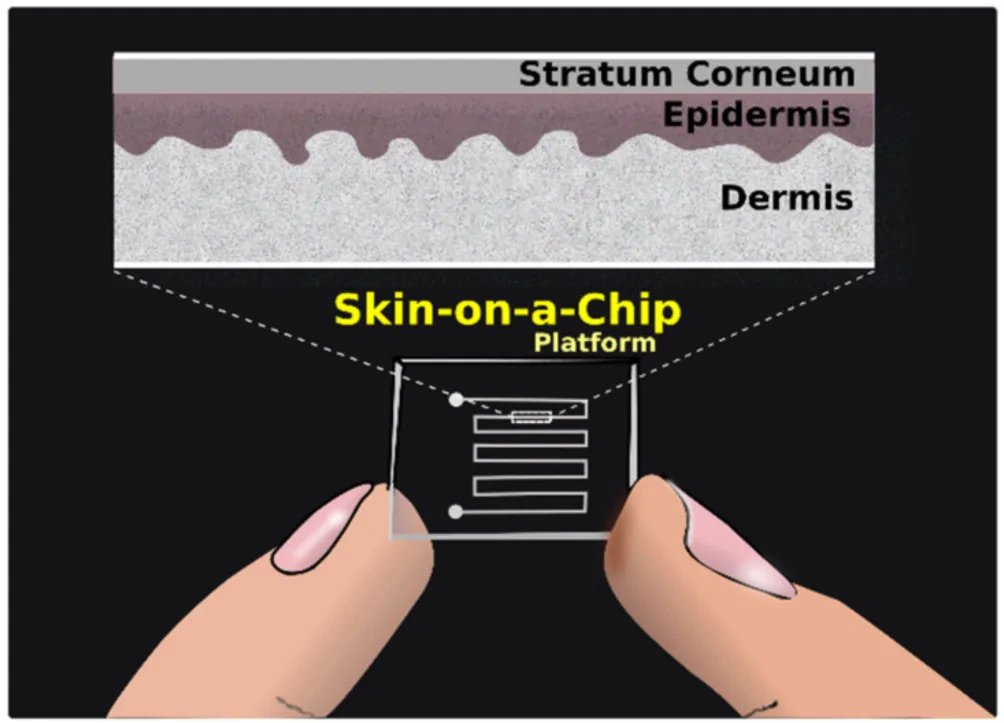
Sketch of a microfluidic skin‐on‐a‐chip platform. Adapted from Ponmozhi et al.,^102^ under CC BY.

Skin explant cultures represent an ex vivo model in which the native spatial organization, cellular composition, and physiological architecture of human skin are preserved for short‐term study. In this approach, full‐thickness or dermis‐level skin sections are maintained under controlled culture conditions, allowing investigation of melanocyte function within an intact tissue context. Using human skin explants, Olivatti et al. demonstrated that melanocytic activity and tissue architecture remain viable for up to 72 h in culture, with increased melanin production observed following ultraviolet and visible light exposure.^20^

The increasing use of engineered skin constructs also raises important ethical considerations. These systems often rely on human‐derived primary cells, underscoring the need for appropriate donor consent, ethical sourcing, and data governance. At the same time, engineered skin and organoid models offer a clear ethical advantage by reducing reliance on animal models, aligning with the principles of Replacement, Reduction, and Refinement (3Rs). Importantly, current pigmentation‐focused skin constructs lack neural integration and sensory capacity, placing them outside ethical concerns associated with sentience. Continued development of these platforms should nevertheless be accompanied by transparent reporting standards and regulatory oversight to ensure responsible translational deployment.^20^

As a highly accessible alternative to actively pumped microfluidic devices, bioengineers have also developed gravity‐driven, pumpless platforms. These simplified systems successfully maintain the viability of full‐thickness skin explanted directly from graft patients, providing a practical ex vivo environment for clinical monitoring and personalized testing.^103^

While custom microfluidic platforms offer excellent physiological simulation, researchers increasingly rely on standardized, commercially available 3D artificial skin models for the high‐throughput screening of therapeutics. Models specifically optimized for melanogenesis research, such as MelanoDerm™, incorporate normal human melanocytes and keratinocytes derived from diverse genetic ancestries (e.g., Black, Asian, or Caucasian donors). These highly differentiated 3D tissue equivalents, alongside other established commercial models like EpiSkin™,^104^ SkinEthic™,^105^ and EpiDerm™,^34^ provide a robust, reproducible testing ground for evaluating the efficacy and safety of novel depigmenting compounds, such as flavonoids and kojic acid.

To achieve even greater structural precision in fabricating these tissue equivalents, the field is rapidly advancing toward 3D bioprinting and specialized step‐wise culture techniques. 3D bioprinting allows for the precise spatial layering of live cells within specialized bioinks to construct complex, customized skin architectures.^106^ By utilizing transwell inserts to establish an air‐liquid interface (ALI), these printed constructs promote proper keratinocyte differentiation, resulting in highly viable, fully stratified skin layers capable of mimicking native tissue.^31^ A brief overview of the skin bioprinting process is depicted in Figure 6, where live cells and structural constituents like gelatin/alginate are incorporated into a bioink. The bioink is extruded using a syringe in a cylindrical shape, using the transwell inserts as a mold. Once printed, the structure is cured with calcium chloride and thrombin. This cross linking hardens the 3D printed skin construct. Immediately after curing, the 3D‐printed skin is kept in a fully submerged culture for 2 days. Finally, the model is transitioned to an ALI culture for 14 days, supplemented with calcium chloride. This air exposure is a critical step, as it forces the keratinocytes to properly differentiate and promote keratinization, ultimately resulting in a highly viable, successfully stratified epidermal layer.^31^

**FIGURE 6:**
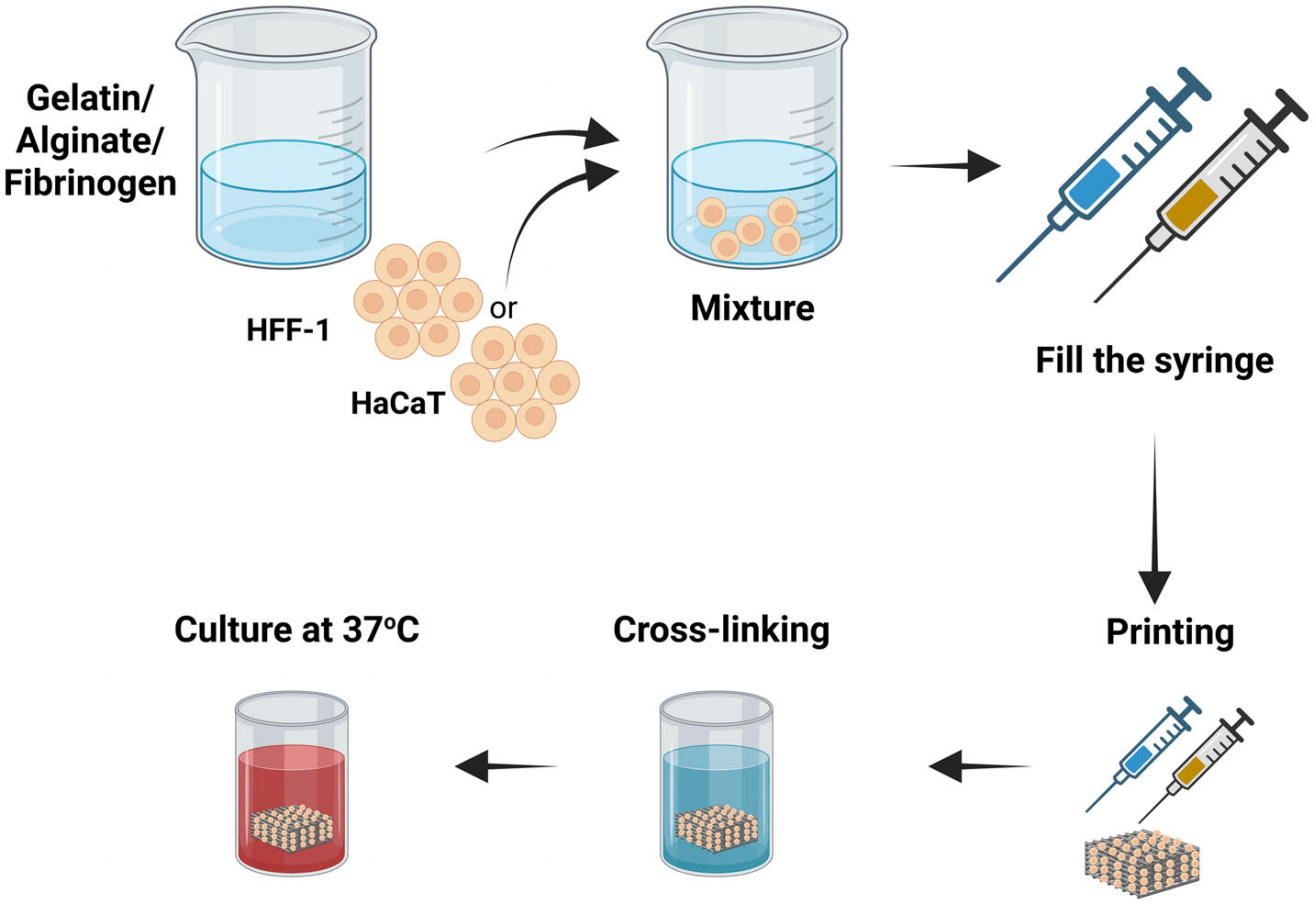
(A) Schematic diagram showing skin printing and ALI culture. Adapted from Jin et al.,^31^ under CC BY 4.0.

Similarly, the generation of Reconstructed Human Pigmented Skin (RHPS) and Reconstructed Human Pigmented Epidermis (RHPE) relies on targeted cellular seeding followed by ALI culture. This methodology successfully yields a functional, pre‐pigmented, stratified epidermis, either resting atop a mature, fibroblast‐populated dermis (RHPS) or isolated on a synthetic supportive membrane (RHPE).^32^, ^33^ By accurately recreating the in vivo epidermal‐melanin unit, these advanced biomimetic systems provide an unparalleled translational pipeline for studying long‐term pigmentation dynamics and evaluating dermatological therapeutics. A visual representation of this step‐wise construction is presented in Figure 7.

**FIGURE 7:**
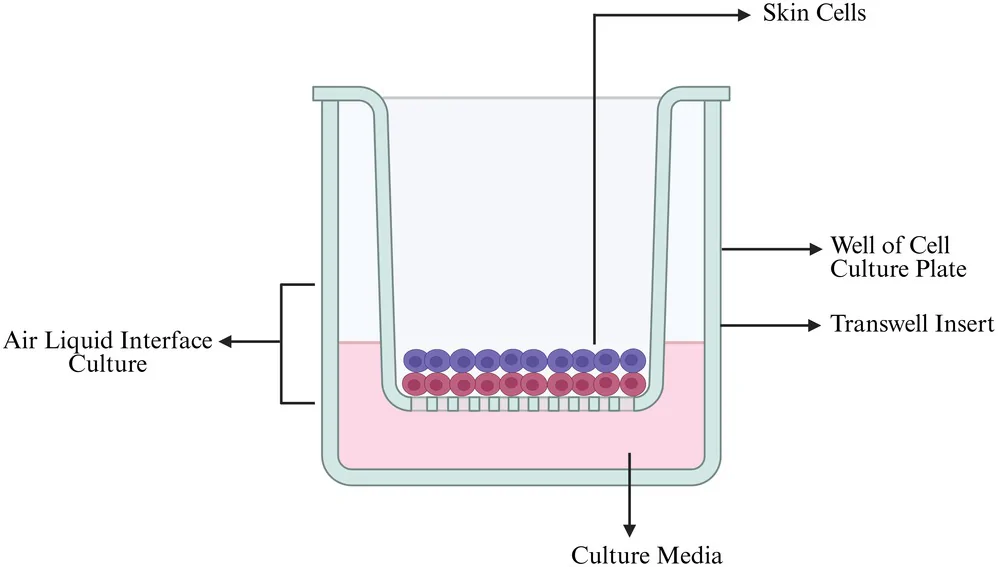
Cells grown in air liquid interface culture. This figure provides a visual representation of Reconstructed Human Pigmented Skin or Epidermis models grown using an Air‐Liquid Interface (ALI). Exposing the apical surface to air is a critical step that drives proper cellular stratification and epidermal keratinisation.

The structural and physiological success of these macroscopic 3D tissue models relies heavily on the air‐liquid interface (ALI) culture technique, which effectively forces the epidermal cells to undergo rapid, accurate stratification and differentiation.^107^ However, while these tissue constructs successfully recreate the cellular microenvironment, bioengineers are also applying synthetic biology at the sub‐cellular level by designing artificial organelles.

Because natural melanogenesis occurs within a highly confined and regulated vesicular environment, creating synthetic melanosomes allows researchers to precisely mimic this localized process for targeted therapeutic applications. For instance, researchers have developed light‐responsive synthetic melanosomes by encapsulating a photoactivatable tyrosinase within lipid‐based vesicles. By design, the enzyme's catalytic activity remains blocked until it is exposed to UV radiation, triggering on‐demand melanin synthesis. When introduced into ex vivo porcine and in vivo mouse skin models, these synthetic organelles successfully provided localized pigmentation and active UV protection, demonstrating their immense potential as programmable, photo‐triggered therapeutics.^39^

Complementing this lipid‐based approach, highly stable artificial melanosomes have also been engineered using specialized polymersomes to encapsulate tyrosinase alongside melanin precursors.^38^ These biomimetic nanoreactors not only demonstrate exceptional long‐term colloidal stability and non‐toxicity to host cells, but they also accurately replicate the natural intracellular behavior of human melanosomes. Upon uptake by keratinocytes, these artificial melanosomes naturally migrate to the perinuclear region, hence perfectly mimicking the UV‐absorbing “nuclear caps” formed by native melanosomes to shield cellular DNA from photodamage.^38^, ^108^ Together, these engineered sub‐cellular platforms highlight how synthetic biology can move beyond merely simulating melanogenesis to actively deploying customizable, synthetic organelles for advanced photoprotection and regenerative dermatology.

The ultimate goal of bioengineered platforms is to advance regenerative medicine beyond traditional surgical grafting techniques by developing fully synthetic, autologous tissue constructs and targeted cell‐free therapeutics. For instance, the development of Reconstructed Human Pigmented Skin (RHPS) and Pigmented Tissue‐Engineered Skin Substitutes (P‐TESSs) using step‐wise air‐liquid interface cultures facilitates the in vitro generation of a functional, pre‐pigmented epidermis atop a mature dermis.^32^, ^108^ Utilizing 3D bioprinting technologies, researchers can incorporate autologous human skin cells into customized bioinks to print these complex, multi‐layered tissue architectures prior to grafting.^31^ In parallel, advanced stem cell technologies offer robust autologous cell sources for restoring pigmentation. Patient‐derived induced pluripotent stem cells (iPSCs) can be differentiated into human iPSC‐derived melanocytes (hiMels) capable of successfully localizing to the basal layer of the epidermis upon transplantation.^108^ Furthermore, novel stem cell sources, such as multilineage‐differentiating stress‐enduring (MUSE) cells isolated from human dermis or adipose tissue, can be directed to differentiate into mature melanocytes and successfully integrate into the basal layer of 3D skin organoids without tumorigenic risks.^109^, ^110^

While biomimetic systems like 3D bioprinted constructs and microfluidic skin‐on‐a‐chip platforms offer unparalleled physiological relevance, their translational feasibility is currently hindered by significant scalability and reproducibility challenges. Fabricating custom 3D architectures or microfluidic devices requires specialized, high‐cost equipment and advanced technical expertise, making them difficult to standardize across different laboratories.^31^, ^102^ Furthermore, although commercial models (e.g., MelanoDerm™) improve reproducibility, they remain expensive and often lack the complete integration of immune cells, vascular networks, and neural components present in native human skin. Consequently, while these systems are excellent for highly predictive, low‐to‐medium throughput localized testing, they possess an inherent translational gap regarding ultra‐high‐throughput screening demands and the modeling of neuro‐immune pigmentary triggers. These engineered constructs offer a transformative regenerative approach; rather than relying solely on traditional grafting techniques, clinicians may soon utilize 3D‐bioprinted, patient‐derived pigmented organoids to seamlessly restore both the structural integrity and the native pigmentation of diseased or damaged skin.

## 6 ENGINEERED MELANIN BIOMATERIALS

Beyond serving as models for studying pigmentary disorders, synthetic biology platforms facilitate the scalable production of melanin as a highly versatile, translatable biomaterial. By decoupling melanin synthesis from human melanocytes, bioengineers can mass‐produce modified melanins for targeted applications in UV protection, antioxidant delivery, and drug transport.

For UV protection and cosmetics, biomimetic approaches have led to the development of artificial melanosomes. By encapsulating tyrosinase and melanin precursors within lipid‐based vesicles^39^ or PDMS‐b‐PMOXA polymersomes,^38^ researchers have created highly stable, biocompatible nanoparticles. These engineered melanosomes not only mimic native cellular distribution but also exhibit potent UV‐absorption properties, offering a translational pathway for developing next‐generation, biologically integrated sunscreens and photoprotective topicals.

Furthermore, microbially synthesized melanin holds significant translational value as an antioxidant delivery system. For instance, the heterologous expression of pyomelanin in yeast systems like Yarrowia lipolytica yields a highly pure pigment with demonstrated photoprotective and antioxidant properties.^94^ When formulated into dermatological treatments, these bioengineered melanins could serve as active vehicles that deliver potent antioxidants directly to the epidermis, mitigating oxidative stress associated with aging or inflammatory skin conditions.

Finally, the unique polymeric and biophysical properties of engineered melanin make it a highly effective drug carrier and functional biomaterial. Because melanin possesses intrinsic metal‐binding and semiconducting properties,^100^ synthetically derived melanins can be modified to act as sophisticated delivery vehicles. A prime translational example is the utilization of synthetic melanins for the chemosorption of radiometals, enabling targeted drug delivery in nuclear medicine and oncology.^111^ Additionally, modular bacterial systems can be utilized to synthesize structurally diverse melanin variants, such as melanin‐diamine complexes,^92^ expanding the repertoire of melanin‐based biomaterials available for both medical and industrial applications.

## 7 CELL‐FREE ASSAYS

Cell‐free assays have emerged as powerful tools to explore complex mechanisms such as melanogenesis in a controlled and simplified environment. Since these are experimental systems that operate outside of living cells, major processes can be studied without interference from extraneous factors. Enzymes, organelles, or biosynthetic extracts are used to delve deeper into the mechanism under study. This method is preferred for testing enzyme kinetics, substrate specificity, action of inhibitors and activators, without the influence of the whole‐cell system.^112^

Cell‐free assays provide a highly controlled, reductionist environment to study melanogenesis without the confounding variables of cell viability, membrane permeability, or complex intracellular signaling networks. For instance, cell‐free tyrosinase assays, typically utilizing mushroom‐derived tyrosinase and L‐DOPA as a substrate, are a cornerstone for high‐throughput pharmacological screening. By isolating the enzymatic reaction, researchers can determine whether a novel depigmenting agent acts as a direct enzymatic inhibitor or functions indirectly through upstream genomic regulation. As a proof of concept, this cell‐free approach was utilized to definitively confirm that plumbagin functions as a direct, potent inhibitor of tyrosinase oxidation, a mechanism that could not be exclusively isolated using traditional live cell lines.^28^ Consequently, these isolated enzymatic assays remain a gold standard for efficiently screening libraries of potential anti‐pigmentary compounds.^113^, ^114^, ^115^

Beyond simple enzymatic screening, advanced synthetic biology tools such as Transcription‐Translation (TXTL) systems allow for the complete in vitro replication of the molecular machinery required to synthesize melanogenic proteins. By utilizing a highly optimized mixture of cell lysates, RNA polymerases, ribosomes, and energy sources, TXTL platforms can transcribe and translate targeted DNA sequences entirely outside of a living host.^30^ Incorporating the genetic sequences for melanogenic enzymes into these TXTL reactions facilitates the rapid, on‐demand biosynthesis of these proteins. This cell‐free expression strategy overcomes the typical biological bottlenecks of heterologous cell cultures, such as host cell toxicity and metabolic resource competition, thereby providing a highly flexible and scalable platform for prototyping novel melanogenic therapeutics and studying pigmentary pathways.

Despite their exceptional reproducibility and scalability for high‐throughput screening, a critical limitation of cell‐free assays is their low physiological relevance. By deliberately isolating enzymatic reactions (e.g., tyrosinase oxidation) from the cellular environment, these assays strip away critical biological barriers.^112^ They cannot predict how a potential therapeutic compound will navigate cell membrane permeability, intracellular degradation, or the complex feedback loops within the native epidermal‐melanin unit. Therefore, while cell‐free platforms possess high translational feasibility for rapid biochemical prototyping and mapping direct enzyme kinetics,^28^ their findings represent an incomplete pharmacological profile and must be subsequently validated in more complex, live‐cell biomimetic models to confirm true physiological efficacy.^113^, ^115^

Finally, cell‐free regenerative approaches are emerging as next‐generation dermatological therapeutics, effectively circumventing the invasive procedures and immunogenic risks associated with whole‐cell transplantation. Acellular therapeutics utilizing platelet‐rich plasma (PRP), extracellular vesicles (EVs), and stem cell conditioned media (CM) or secretomes deliver highly concentrated, targeted bioactive molecules, including mitogenic growth factors and ROS‐scavenging antioxidants.^109^, ^116^ By modulating the entire pathological microenvironment rather than just replacing melanocytes, these cell‐free systems stimulate resident melanocyte precursors, counteract oxidative stress, and promote deep tissue regeneration, offering a transformative, non‐invasive approach for treating complex depigmenting disorders like vitiligo.^108^, ^109^

A general overview of these cell‐free methodologies is represented in Figure 8.

**FIGURE 8:**
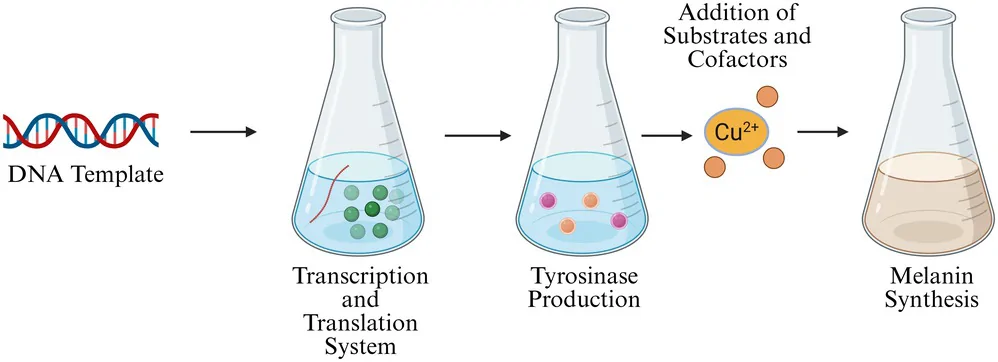
An overview of the cell free assays in melanogenesis research.

All the systems mentioned in this review have their own merits and demerits, which have been catalogued in Table 4.

**TABLE 4:** An overview of the various systems employed for studies on melanogenesis and pigmentation.

Sl. No.	Type of System	Sub Type	Advantage(s)	Disadvantage(s)	Best suited use cases	Citation(s)
1.	Genetic Engineering	CRISPR‐Cas9	Enables disease relevant genetic manipulations with high precision. Can be used for functional validation of gene mutations. Can be used as a somatic therapy tool in pigmentary disorders.	Potential for off‐target edits at unintended sites. Delivering the CRISPR‐Cas9 components to the target cells can be challenging.	Mechanistic studies; pathway dissection; target validation	75, 80
		TALENs	Less possibility of off‐target edits. Can be used to disrupt specific genes.	Protein engineering for TALENs is complex.		29, 74
2.	Heterologous Systems	Engineered Microbes	Enables the expression of genes and proteins from one species in another. Facilitates large scale melanin production for commercial applications.	The expressed proteins may not function in the same way as the native protein does.	Enzyme screening; pigment biosynthesis; industrial biotechnology	90, 93
3.	Biomimetic Systems	Co‐cultures	Co‐cultures of melanocytes and keratinocytes better depict the normal human skin conditions.	Difficult to maintain and standardize in comparison to normal cells.	Preclinical compound screening; UV‐response assessment	37
		Spheroid Culture	Promotes higher melanin production compared to monolayer cultures.	More difficult to maintain than normal cells yet lack the cellular complexity of human skin.	3D pigmentation dynamics; diffusion‐limited compound screening	36
		Organoids	Organoids retain the physiology of human skin in were reported by approximately 50% of respondents in a large multinational survey vitro conditions.	Creation and maintenance of this system is complex.	Ex vivo topical efficacy and UV‐response assessment	20
		Organ‐on‐chip	This model can mimic physiological conditions, blood flow and nutrient exchange	Creation and maintenance of this system is complex.	Predictive toxicity testing; chronic exposure studies	35
		3D Bio‐printed Systems	Reconstructs the layers of human skin, and allows for the creation of standard, reproducible models for disease modeling and drug screening.	The process is expensive, and requires specialized printers with bioink.	Spatially controlled pigmentation models; preclinical compound screening	31
4.	Cell‐free assays	Enzyme Assays	Provides an ideal environment to study the action of enzymes, substrates, drugs or other compounds without cellular interference.	The lack of complexity may not capture the full biological effect of a compound.	Early‐stage inhibitor screening; assay development	28
		TXTL Systems	This system allows for the production of biomolecules outside the cell, which provides a low cost alternative to synthesize molecules without the need for cell culture.	The yield of the biomolecule can vary significantly. The cell lysate used can contain endogenous enzymes that can interfere with the needed reaction.	Rapid enzyme prototyping and high‐throughput biochemical screening	30

The information regarding the translational use cases and the primary experimental readout of each of the systems provided in this paper is explained in Table 4. A comprehensive critical evaluation comparing the physiological relevance, scalability, and translational limitations of all the engineered platforms discussed in this review is provided in the summary matrix (refer to Table 5).

**TABLE 5:** Comprehensive mapping and critical evaluation of engineered melanogenesis platforms.

Sl. No.	Model platform	Primary experimental readout	Representative translational use—Cases	Physiological relevance	Reproducibility and scalability	Limitations and translational gaps	References
1.	Genetic Engineering (CRISPR‐Cas9, TALENs)	Mutation correction efficiency, Melanin content, tyrosinase activity, MITF signaling, pigmentation phenotypes, transcriptomic	Pathway‐specific target validation for vitiligo re‐pigmentation and hyperpigmentation disorders; mechanistic screening of pigmentation modulators	High (when applied to human cells), allows exact correction or induction of human disease mutations.	Low scalability for mass production, High reproducibility for specific gene knockouts.	High potential for unintended off‐target edits; challenges with long‐term stability and delivery efficiency in continuously renewing tissue.	75, 76, 78, 80
2.	Biomimetic skin equivalents / 3D reconstructed epidermis	Pigment distribution, melanosome transfer efficiency, UV‐induced pigmentation responses, permeation and penetration of depigmenting agents	Preclinical screening of topical depigmenting or photoprotective agents; assessment of UV response and pigmentation heterogeneity	Very High, accurately replicates 3D epidermal architecture, air‐liquid interface, and paracrine signaling.	Moderate, standardized commercial models exist, but custom bioprinting is expensive and complex.	High cost and technical complexity, constructs currently lack neural integration and active immune cell components.	20, 31, 32, 36, 37
3.	Skin‐on‐chip/organ‐on‐chip platforms	Dynamic pigmentation changes, barrier integrity, inflammatory and stress markers	Predictive safety and toxicity testing; long‐term exposure and UV‐response studies under physiologically relevant conditions	High; introduces systemic factors like blood flow, oxygen gradients, and circulating hormones.	Low to Moderate, requires specialized microfluidic setups and complex maintenance.	Difficult to standardize across different laboratories, high technical threshold for routine high‐throughput screening.	35, 102
4.	Heterologous expression systems (yeast, bacterial hosts)	Enzyme activity, reaction throughput, pigment yield and composition	High‐throughput screening of tyrosinase inhibitors; scalable production of melanin or melanin intermediates for industrial applications	Very Low, lacks melanosomes, eukaryotic post‐translational modifications, and intercellular signaling.	Extremely High, enables rapid, industrial‐scale mass production of pigment.	Poor representation of human melanocyte biology, expressed mammalian proteins may not fold or function natively.	27, 90, 92
5.	Cell‐free and in vitro melanogenesis assays	Enzyme kinetics, inhibitor IC_50_ values, melanin polymerization rates	Rapid screening of cosmetic and therapeutic inhibitors; assay development for diagnostic or quality‐control applications	Low, deliberately isolates enzymes away from cellular networks and feedback loops.	High, easily standardized for rapid, high‐throughput biochemical screening.	Complete lack of cellular complexity, cannot predict drug behavior against cell membrane permeability or intracellular regulation.	28, 30, 115

## 8 MAPPING ENGINEERED TOOLS TO MELANIN PRODUCTION PATHWAYS

### 8.1 Targeting specific genes using genetic engineering

Genetic editing tools like CRISPR‐Cas9 are mostly used to intentionally alter specific genes inside the cell to see exactly how they control the melanin‐making chain reactions. For example, instead of relying on naturally occurring conditions, researchers use genetic modifications to intentionally turn off fundamental genes, such as the *MC1R* receptor, the *TYR* gene, or the *OCA2* gene. By breaking these specific links, they can deliberately block melanin production and study the resulting loss of skin color under highly controlled conditions.^55^ On the other hand, genetic tools can also be used to reverse these effects by repairing broken pathways. For instance, in models of severe albinism, CRISPR was used to correct a specific genetic typo (the T373K mutation). Fixing this single genetic target successfully restarted the tyrosinase enzyme (the main enzyme that makes melanin) and dramatically boosted melanin production.^78^ Similarly, other gene‐editing tools like TALENs have been used to break the *slc24a5* gene, directly resulting in a drop in melanin and pale patches on the body.^29^ By linking exact gene edits directly to the cell's melanin‐making machinery, researchers can pinpoint the precise targets to treat skin pigmentation disorders.

### 8.2 Preserving cell communication and feedback loops in biomimetic systems

Basic cells grown in a dish often lose their ability to communicate with each other. However, advanced biomimetic systems are specially built to keep the natural “feedback loops” (cause‐and‐effect communication) active between skin cells. In real skin, making melanin relies heavily on cells sending chemical messages to each other. For example, a molecule called α‐MSH tells the cell to start the chain reactions that make melanin.^45^ Basic single‐cell cultures completely lose this cell‐to‐cell chatter. But by building 3D bioprinted skin and lab‐grown human skin models that have a natural air‐liquid boundary, engineers bring back this crucial communication. This lets researchers study how melanin is naturally made and shared between cells.^31^, ^32^ Additionally, using microfluidic “skin‐on‐a‐chip” models allows researchers to pump hormones into the system. For instance, pumping the hormone 17β‐estradiol through the chip activates estrogen pathways that trigger melanin production. This perfectly mimics the hormone‐driven dark spots (melasma) that often appear during pregnancy.^35^

### 8.3 Isolating reactions using cell‐free systems

While biomimetic models try to keep complex cell communication alive, cell‐free systems do the exact opposite. They are designed to intentionally remove all cell communication so researchers can study one isolated reaction. When testing new skin‐lightening drugs on whole cells, it is hard to tell if the drug is stopping the early chemical signals (like the MAPK/CREB pathway) or if it is directly blocking the enzyme that makes melanin.^114^, ^115^ Cell‐free tools solve this by taking the melanin‐making enzyme (tyrosinase) completely out of the cell, away from all the confusing background signals.^112^ This separation lets researchers see clearly if a new drug works by directly attacking the enzyme or by changing the earlier cell signals. For example, this method was used to prove that a compound called plumbagin works by directly blocking the tyrosinase enzyme from functioning.^28^ A complete hierarchical overview of how these distinct tools are applied across varying levels of biological complexity is illustrated in our multi‐scale model (refer to Figure 9).

**FIGURE 9:**
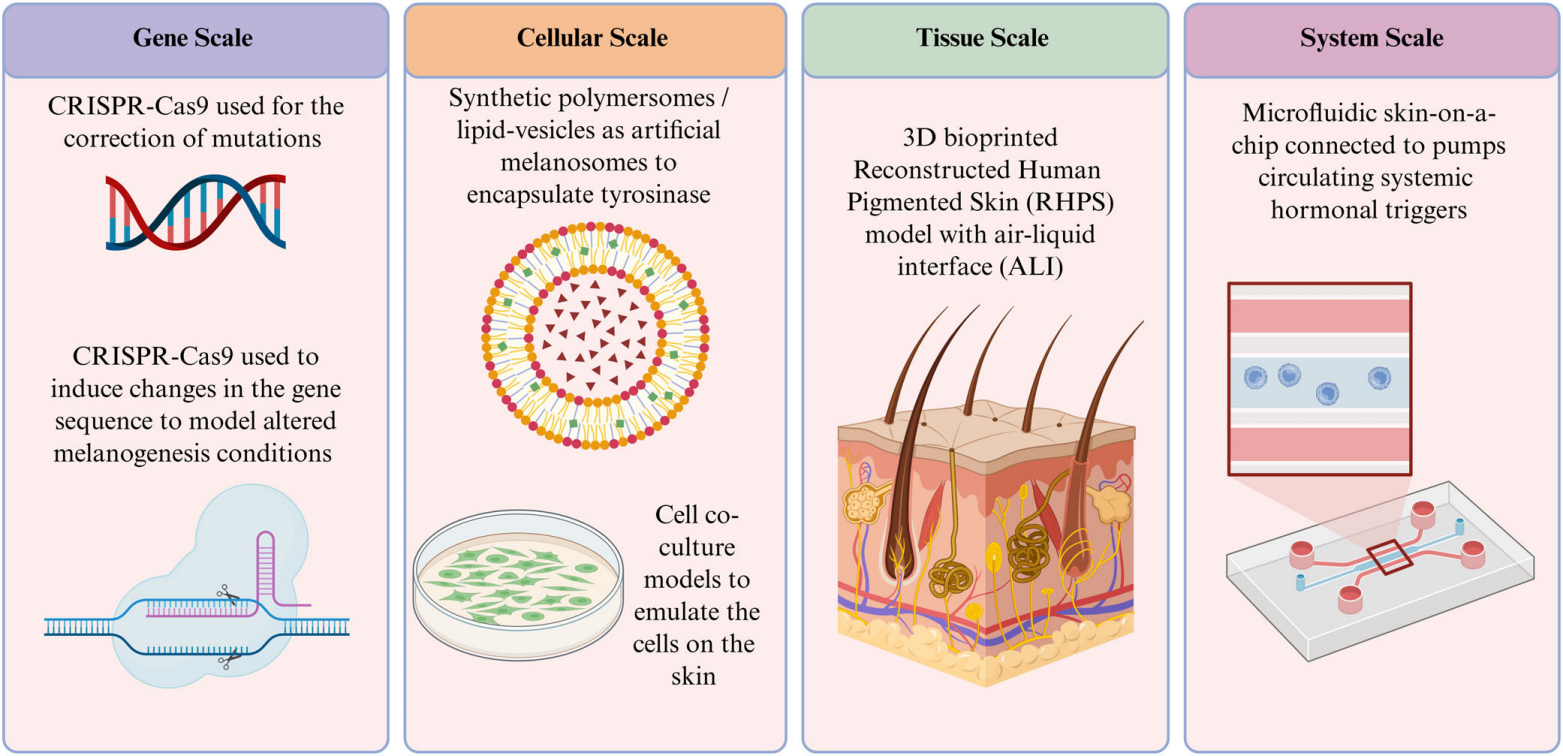
Multi‐scale modeling of engineered pigmentation systems.

## 9 CONCLUSION

Synthetic biology has ushered in a new era of dermatological research, enabling the elucidation of molecular mechanisms and screening of potential therapeutics in a rapid, precise, cost‐effective and ethical manner. Genetic engineering offers precision, reproducibility, and control that traditional methods cannot. Biomimetic systems help replicate the skin architecture and offer a customizable platform for drug screening and studies on the effect of environmental stimuli on the skin. Heterologous systems enable the production of melanin in organisms that don't generally produce the pigment but are suitable for efficient growth and study in the lab environment. Cell‐free assays are useful in cases where a simplified system to only study certain parts of the process is necessary. These methods enable the creation of disease‐specific models, industrial‐scale melanin production systems, or the simulation of pigmentary conditions without relying solely on naturally occurring variants. From microbial platforms expressing melanogenic genes to cell‐free enzymatic assays and sophisticated 3D skin constructs, these tools collectively allow for a modular, customizable, and ethically sound investigation into pigmentation biology. These systems not only facilitate a deeper understanding of the expression of pigmentation of the skin but can also produce melanin for use as dyes in the textile industry, UV protectant in cosmetic products, binding compound for bioremediation of contaminated sites, and as organic semiconductors in electronics. These synthetic systems offer unprecedented precision in dissecting the regulatory networks and enzymatic cascades that govern melanin biosynthesis, while also providing scalable platforms for screening pigmentation modulators and understanding disease pathophysiology (refer to Figure 10).

**FIGURE 10:**
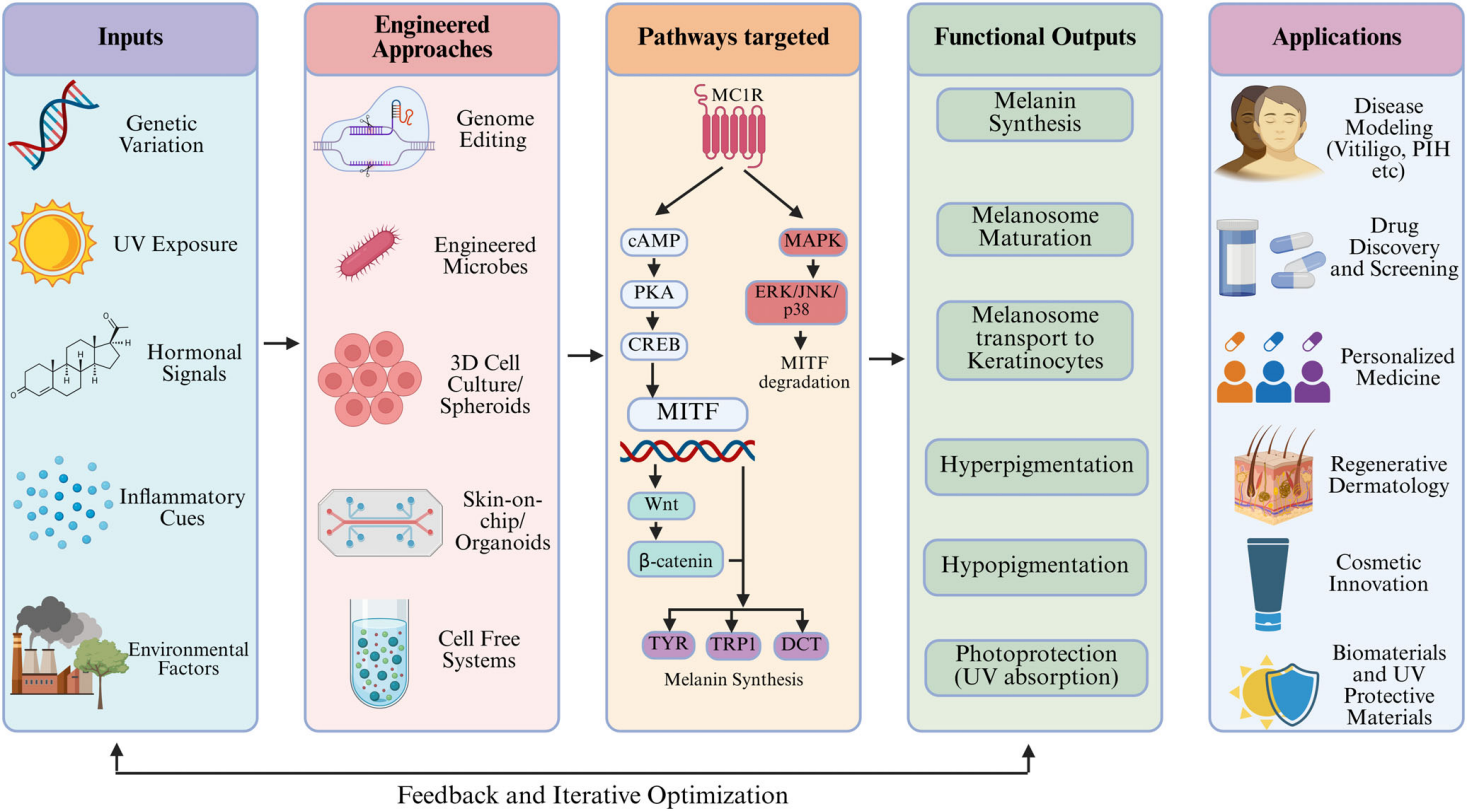
Comprehensive workflow for engineered pigmentation systems. This framework maps how clinical and environmental inputs are investigated using distinct synthetic biology tools. These platforms target key melanogenesis pathways to measure functional outputs, such as melanin synthesis and melanosome transport. Through continuous iterative feedback, these systems converge into actionable translational applications, including regenerative dermatology, high‐throughput drug screening, and the development of novel biomaterials.

## 10 CHALLENGES AND FUTURE PERSPECTIVES

The successful translation of synthetic biology platforms into clinical therapeutics and cosmeceutical products introduces distinct technological challenges and necessitates strict adherence to evolving regulatory frameworks. To fully realize their clinical potential, several key barriers must be addressed.

### 10.1 Delivery systems for skin applications

A major hurdle in translating CRISPR‐Cas9 therapies for pigmentary disorders is effectively delivering the genome‐editing components across the highly protective human stratum corneum. Traditional viral vectors, such as adeno‐associated viruses (AAVs), are frequently used in gene therapy but possess a restricted cargo capacity (~4.7 kb) that complicates the packaging of the large Cas9 sequence alongside the sgRNA.^79^ Furthermore, lentiviral vectors carry the inherent risk of integrating into the host genome, potentially leading to insertional mutagenesis.^79^ To overcome these limitations, recent advancements have focused on non‐viral, localized delivery systems specifically tailored for the skin. For example, the transdermal delivery of CRISPR‐Cas9 ribonucleoproteins (RNPs) targeting melanocytes has been successfully achieved using polyamines‐modified thermosensitive hydrogels. These vehicles remain fluid at room temperature for application but form a stable gel at body temperature, allowing for the sustained, localized release of gene‐editing components without systemic toxicity.^81^ Similarly, lipid‐based nanoparticles (LNPs) and extracellular vesicles (EVs) are emerging as highly biocompatible carriers capable of fusing with target cell membranes to deliver CRISPR cargos efficiently, transiently, and with low immunogenicity.^79^

### 10.2 Safety concerns: immunogenicity and tumorigenicity

Even with successful delivery, the safety profile of genetic tools like CRISPR‐Cas9 must be rigorously evaluated. Because Cas9 proteins are primarily derived from bacteria (e.g., *Streptococcus pyogenes* or *Staphylococcus aureus*), the human immune system frequently recognizes them as foreign antigens, triggering robust immune responses that can reduce therapeutic efficacy and complicate repeated treatments.^79^ Additionally, the risk of off‐target effects remains a critical concern; unintended DNA cleavage can result in genomic rearrangements, immune reactions, and potentially tumorigenesis via oncogene activation.^79^ Mitigating these tumorigenic risks requires the continued optimization of high‐fidelity Cas9 variants, the use of truncated sgRNAs to increase binding specificity, and the implementation of anti‐CRISPR (Acr) proteins to precisely control nuclease activity and prevent unwanted cuts.^79^

### 10.3 Regulatory barriers and ethical considerations

To navigate the regulatory aspects related to these novel therapeutics and cosmeceuticals, the industry is increasingly relying on New Approach Methodologies (NAMs).^117^ Following guidelines from the Organization for Economic Cooperation and Development (OECD), 3D reconstructed human epidermis models have been formally accepted by the FDA and the Center for Drug Evaluation and Research (CDER) for dermal irritation and phototoxicity testing.^117^ Consequently, these bioengineered models can now directly support safety demonstrations for Investigational New Drug (IND) submissions and cosmeceutical approvals, providing a clear regulatory pathway that aligns with the ethical 3Rs (Replacement, Reduction, and Refinement) of animal research. However, the industrialization of these therapies, particularly engineered live therapeutics (ELTs), falls under the strict regulatory purview of the FDA's Centre for Biologics Evaluation and Research (CBER), requiring rigorous chemistry, manufacturing, and control standards, as well as complex pharmacokinetic tracking.^118^

The use of engineered skin constructs also introduces ethical and regulatory considerations regarding the sourcing of human‐derived primary cells, underscoring the need for appropriate donor consent, transparent reporting standards, and strict data governance before these models can be responsibly deployed for translational screening. Furthermore, evaluating these clinical therapeutics and cosmetic products increasingly requires objective standardization to satisfy regulatory bodies. Recent FDA safety communications have highlighted the need to resolve racial disparities in the performance of optical medical devices and therapeutics, underscoring the regulatory necessity of using standardized, objective tools like melanometry to precisely quantify skin pigmentation across diverse populations during clinical validation.^119^

### 10.4 Industrial feasibility of melanin production platforms

Finally, the industrialization of heterologous microbial systems for the mass production of melanin and its derivatives for cosmetics and pharmaceuticals necessitates strict biosafety oversight. While engineered hosts like *Vibrio natriegens* offer exceptionally rapid melanin production,^27^ mitigating the risks of environmental release and horizontal gene transfer is a regulatory imperative. Industrial‐scale facilities must employ robust biosafety frameworks, including physical containment, the utilization of auxotrophic or non‐pathogenic host strains, and engineered genetic safeguards. This ensures that the large‐scale biomanufacturing of synthetic biology‐derived cosmeceuticals remains both scalable, safe, and compliant with environmental regulations.

## AUTHOR CONTRIBUTIONS

**Usha Y. Nayak:** Writing – review and editing; supervision. **B. Anika:** Conceptualization; investigation; writing – original draft; writing – review and editing; data curation. **B. S. Swarna:** Writing – review and editing; supervision. **Vijendra Prabhu:** Writing – review and editing; supervision. **A. S. Bharath Prasad:** Writing – review and editing; supervision; conceptualization. **Arshia Tarkunde:** Writing – review and editing; supervision; conceptualization. **Raghavendra Rao:** Writing – review and editing; supervision.

## FUNDING INFORMATION

No funding was received to assist with the preparation of this manuscript.

## CONFLICT OF INTEREST STATEMENT

The authors have no conflict of interest relevant to this article to disclose.

## CONSENT FOR PUBLICATION

All authors give consent for publication.

## Data Availability

The figures used in this review are eligible for reprinting through the Creative Commons License (CCBY).

## References

[1] Passeron T , Liu W , Morita A , et al. Pigmentary disorders around the world: self‐reported prevalence and impact on QOL and social stigmatization. J Invest Dermatol. 2026;146(6):1661‐1668.e5. doi:10.1016/j.jid.2025.10.598 41203010

[2] Hartmann A . Back to the roots: die Anfänge der Dermatologie in der altägyptischen Medizin. JDDG J Ger Soc Dermatol. 2016;14(4):389‐396. doi:10.1111/ddg.12947 27027749

[3] Kuldeep , Verma S , Awasthi S , Vikas . An overview of Kustha and its management approaches in Ayurvedic texts. J Ayurveda Integr Med Sci. 2023;8(6):91‐96. doi:10.21760/jaims.8.6.15

[4] Blume‐Peytavi U , Bagot M , Tennstedt D , et al. Dermatology today and tomorrow: from symptom control to targeted therapy. J Eur Acad Dermatol Venereol. 2019;33:3‐36. doi:10.1111/jdv.15335 30561009

[5] Henrique Franco N . Animal experiments in biomedical research: a historical perspective. Animals (Basel). 2013;3(1):238‐273. doi:10.3390/ani3010238 PMC4495509 26487317

[6] Serre C , Busuttil V , Botto JM . Intrinsic and extrinsic regulation of human skin melanogenesis and pigmentation. Int J Cosmet Sci. 2018;40(4):328‐347. doi:10.1111/ics.12466 29752874

[7] Brenner M , Hearing VJ . The protective role of melanin against UV damage in human skin. Photochem Photobiol. 2008;84(3):539‐549. doi:10.1111/j.1751-1097.2007.00226.x PMC2671032 18435612

[8] D'Arcy C , Bass O , Junk P , et al. Disease–gene networks of skin pigmentation disorders and reconstruction of protein–protein interaction networks. Bioengineering (Basel). 2023;10(1):13. doi:10.3390/bioengineering10010013 PMC9854651 36671585

[9] Wang F , Ma W , Fan D , Hu J , An X , Wang Z . The biochemistry of melanogenesis: an insight into the function and mechanism of melanogenesis‐related proteins. Front Mol Biosci. 2024;11:1440187. doi:10.3389/fmolb.2024.1440187 PMC11368874 39228912

[10] Schallreuter KU , Wazir U , Kothari S , Gibbons NCJ , Moore J , Wood JM . Human phenylalanine hydroxylase is activated by H 2O 2: a novel mechanism for increasing the L‐tyrosine supply for melanogenesis in melanocytes. Biochem Biophys Res Commun. 2004;322(1):88‐92. doi:10.1016/j.bbrc.2004.07.082 15313177

[11] Schallreuter KU , Wood JM . The Importance of L‐Phenylalanine Transport and Its Autocrine Turnover to L‐Tyrosine for Melanogenesis in Human Epidermal Melanocytes. 1999 http://www.idealibrary.com 10.1006/bbrc.1999.1241 10462491

[12] Snyman M , Walsdorf RE , Wix SN , Gill JG . The metabolism of melanin synthesis—from melanocytes to melanoma. Pigment Cell Melanoma Res. 2024;37(4):438‐452. doi:10.1111/pcmr.13165 PMC11178461 38445351

[13] Újvári A , Aron R , Eisenhaure T , et al. Translation rate of human Tyrosinase determines its N‐linked glycosylation level. J Biol Chem. 2001;276(8):5924‐5931. doi:10.1074/jbc.M009203200 11069924

[14] Atwood SX , Plikus M . Fostering a healthy culture: biological relevance of in vitro and ex vivo skin models. Exp Dermatol. 2021;30(3):298‐303. doi:10.1111/exd.14296 PMC8142902 33565670

[15] Benito‐Martínez S , Zhu Y , Jani RA , Harper DC , Marks MS , Delevoye C . Research techniques made simple: cell biology methods for the analysis of pigmentation. J Invest Dermatol. 2020;140(2):257‐268.e8. doi:10.1016/j.jid.2019.12.002 PMC6986772 31980058

[16] Alcantara GP , Esposito ACC , Olivatti TOF , Yoshida MM , Miot HA . Evaluation of ex vivo melanogenic response to UVB, UVA, and visible light in facial melasma and unaffected adjacent skin. An Bras Dermatol. 2020;95(6):684‐690. doi:10.1016/j.abd.2020.02.015 PMC7672495 33010989

[17] Qu J , Yan M , Fang Y , et al. Zebrafish in dermatology: a comprehensive review of their role in investigating abnormal skin pigmentation mechanisms. Front Physiol. 2023;14:1296046. doi:10.3389/fphys.2023.1296046 PMC10702362 38074315

[18] Sun X , Wang W , Yang H , et al. A comparative study on the sensitivity of establishing melasma‐like models in different strains of mice. J Cosmet Dermatol. 2025;24(4):e70155. doi:10.1111/jocd.70155 PMC11956124 40162532

[19] Cooper CD . Insights from zebrafish on human pigment cell disease and treatment. Dev Dyn. 2017;246(11):889‐896. doi:10.1002/dvdy.24550 28710811

[20] Olivatti TOF , Alcantara GP , Lemos ACCE , da Silva MG , Miot HA . Standardization of organoid culture for evaluation of melanogenesis induced by UVB, UVA and visible light. An Bras Dermatol. 2020;95(1):46‐51. doi:10.1016/j.abd.2019.06.005 PMC7058865 31901368

[21] Subburaj K , Manjunath S , Kumaran MS . Animal models in disorders of skin color: utilizing evolution and demystifying mysteries. Pigment Int. 2019;6(1):9.

[22] Baxter LL , Pavan WJ . The etiology and molecular genetics of human pigmentation disorders. Wiley Interdiscip Rev Dev Biol. 2013;2(3):379‐392. doi:10.1002/wdev.72 PMC3694277 23799582

[23] Tan X , Letendre JH , Collins JJ , Wong WW . Synthetic biology in the clinic: engineering vaccines, diagnostics, and therapeutics. Cell. 2021;184(4):881‐898. doi:10.1016/j.cell.2021.01.017 PMC7897318 33571426

[24] Cameron DE , Bashor CJ , Collins JJ . A brief history of synthetic biology. Nat Rev Microbiol. 2014;12(5):381‐390. doi:10.1038/nrmicro3239 24686414

[25] Liu AP , Appel EA , Ashby PD , et al. The living interface between synthetic biology and biomaterial design. Nat Mater. 2022;21(4):390‐397. doi:10.1038/s41563-022-01231-3 PMC10265650 35361951

[26] Li T , Yang Y , Qi H , et al. CRISPR/Cas9 therapeutics: progress and prospects. Signal Transduct Target Ther. 2023;8(1):36. doi:10.1038/s41392-023-01309-7 PMC9841506 36646687

[27] Wang Z , Tschirhart T , Schultzhaus Z , et al. Melanin produced by the fast‐growing marine bacterium *Vibrio natriegens* through heterologous biosynthesis: characterization and application. Appl Environ Microbiol. 2020;86(5):e02749‐19. doi:10.1128/AEM.02749-19 PMC7028964 31836580

[28] Oh TI , Yun JM , Park EJ , Kim YS , Lee YM , Lim JH . Plumbagin suppresses α‐MSH‐induced melanogenesis in B16F10 mouse melanoma cells by inhibiting tyrosinase activity. Int J Mol Sci. 2017;18(2):320. doi:10.3390/ijms18020320 PMC5343856 28165370

[29] Pandey D , Matsubara T , Saito T , et al. Talen‐mediated gene editing of slc24a5 (solute carrier family 24, member 5) in kawakawa, euthynnus affinis. J Mar Sci Eng. 2021;9(12):1378. doi:10.3390/jmse9121378

[30] Garenne D , Noireaux V . Cell‐free transcription–translation: engineering biology from the nanometer to the millimeter scale. Curr Opin Biotechnol. 2019;58:19‐27. doi:10.1016/j.copbio.2018.10.007 30395952

[31] Jin S , Oh YN , Son YR , et al. Three‐dimensional skin tissue printing with human skin cell lines and mouse skin‐derived epidermal and dermal cells. J Microbiol Biotechnol. 2022;32(2):238‐247. doi:10.4014/jmb.2111.11042 PMC9628848 34949744

[32] Hall MJ , Lopes‐Ventura S , Neto M v , et al. Reconstructed human pigmented skin/epidermis models achieve epidermal pigmentation through melanocore transfer. Pigment Cell Melanoma Res. 2022;35(4):425‐435. doi:10.1111/pcmr.13039 PMC9543140 35325505

[33] Zoio P , Ventura S , Leite M , Oliva A . Pigmented full‐thickness human skin model based on a fibroblast‐derived matrix for long‐term studies. Tissue Eng C Methods. 2021;27(7):433‐443. doi:10.1089/ten.tec.2021.0069 PMC8309425 34148380

[34] Netzlaff F , Lehr CM , Wertz PW , Schaefer UF . The human epidermis models EpiSkin®, SkinEthic® and EpiDerm®: An evaluation of morphology and their suitability for testing phototoxicity, irritancy, corrosivity, and substance transport. Eur J Pharm Biopharm. 2005;60(2):167‐178. doi:10.1016/j.ejpb.2005.03.004 15913972

[35] Jeong U , Yoon S , Park S , Jeon TJ , Kim SM . 3D artificial skin platform for investigating pregnancy‐related skin pigmentation. Micromachines (Basel). 2024;15(4):511. doi:10.3390/mi15040511 PMC11052160 38675322

[36] Zurina IM , Gorkun AA , Dzhussoeva E , et al. Human melanocyte‐derived spheroids: a precise test system for drug screening and a multicellular unit for tissue engineering. Front Bioeng Biotechnol. 2020;8:540. doi:10.3389/fbioe.2020.00540 PMC7287162 32582665

[37] Kumar R , Parsad D , Kanwar A , Kaul D . Development of melanocye‐keratinocyte co‐culture model for controls and vitiligo to assess regulators of pigmentation and melanocytes. Indian J Dermatol Venereol Leprol. 2012;78(5):599‐604. doi:10.4103/0378-6323.100567 22960816

[38] Meyer CE , Schoenenberger CA , Wehr RP , Wu D , Palivan CG . Artificial melanogenesis by confining melanin/polydopamine production inside polymersomes. Macromol Biosci. 2021;21(12):e2100249. doi:10.1002/mabi.202100249 34510748

[39] Lee UJ , Ko J , Kim SH , et al. Light‐triggered in situ biosynthesis of artificial melanin for skin protection. Adv Sci (Weinh). 2022;9(7):2103503. doi:10.1002/advs.202103503 PMC8895148 34989175

[40] Peng X , Ma Y , Yan C , et al. Mechanism, formulation, and efficacy evaluation of natural products for skin pigmentation treatment. Pharmaceutics. 2024;16(8):1022. doi:10.3390/pharmaceutics16081022 PMC11359997 39204367

[41] Abdo JM , Sopko NA , Milner SM . The applied anatomy of human skin: a model for regeneration. Wound Med. 2020;28:100179. doi:10.1016/j.wndm.2020.100179

[42] Bento‐Lopes L , Cabaço LC , Charneca J , Neto M v , Seabra MC , Barral DC . Melanin's journey from melanocytes to keratinocytes: uncovering the molecular mechanisms of melanin transfer and processing. Int J Mol Sci. 2023;24(14):11289. doi:10.3390/ijms241411289 PMC10379423 37511054

[43] Bao M , Gempeler M , Campiche R . Melanosome transport and processing in skin pigmentation: mechanisms and targets for pigmentation modulation. Int J Mol Sci. 2025;26(17):8630. doi:10.3390/ijms26178630 PMC12429606 40943549

[44] Fowlkes N , Clemons K , Rider PJF , et al. Factors affecting growth kinetics and spontaneous metastasis in the B16F10 syngeneic murine melanoma model. Comp Med. 2019;69(1):48‐54. doi:10.30802/AALAS-CM-18-000036 PMC6382051 30563585

[45] Bahraman AG , Jamshidzadeh A , Keshavarzi M , Arabnezhad MR , Mohammadi H , Mohammadi‐Bardbori A . α‐Melanocyte‐stimulating hormone triggers melanogenesis via activation of the aryl hydrocarbon receptor pathway in B16F10 mouse melanoma cells. Int J Toxicol. 2021;40(2):153‐160. doi:10.1177/1091581820987548 33438493

[46] Rafiq RA , Quadri A , Nazir LA , Peerzada K , Ganai BA , Tasduq SA . A potent inhibitor of phosphoinositide 3‐kinase (PI3K) and mitogen activated protein (MAP) kinase signalling, quercetin (3, 3′, 4′, 5, 7‐pentahydroxyflavone) promotes cell death in ultraviolet (UV)‐B‐irradiated B16F10 melanoma cells. PLoS One. 2015;10(7):e0131253. doi:10.1371/journal.pone.0131253 PMC4493061 26148186

[47] Ferreira IG , Weber MB , Bonamigo RR . History of dermatology: the study of skin diseases over the centuries. An Bras Dermatol. 2021;96(3):332‐345. doi:10.1016/j.abd.2020.09.006 PMC8178570 33814211

[48] Morais MCC , Stuhl I , Sabino AU , et al. Stochastic model of contact inhibition and the proliferation of melanoma in situ. Sci Rep. 2017;7(1):8026. doi:10.1038/s41598-017-07553-6 PMC5556068 28808257

[49] Perdomo J , Quintana C , González I , et al. Melatonin induces melanogenesis in human SK‐MEL‐1 melanoma cells involving glycogen synthase kinase‐3 and reactive oxygen species. Int J Mol Sci. 2020;21(14):1‐16. doi:10.3390/ijms21144970 PMC7404125 32674468

[50] Daveri E , Valacchi G , Romagnoli R , Maellaro E , Maioli E . Antiproliferative effect of Rottlerin on Sk‐Mel‐28 melanoma cells. Evid Based Complement Alternat Med. 2015;2015:1‐9. doi:10.1155/2015/545838 PMC4464680 26161122

[51] Jeon SJ , Choi EY , Han EJ , et al. Piperlongumine induces apoptosis via the MAPK pathway and ERK‐mediated autophagy in human melanoma cells. Int J Mol Med. 2023;52(6):115. doi:10.3892/ijmm.2023.5318 PMC10599349 37830157

[52] Bajpai VK , Swigut T , Mohammed J , et al. A genome‐wide genetic screen uncovers determinants of human pigmentation. Science. 2023;381(6658):eade6289. doi:10.1126/science.ade6289 PMC10901463 37561850

[53] Ma LP , Liu MM , Liu F , et al. Melatonin inhibits senescence‐associated melanin pigmentation through the p53‐TYR pathway in human primary melanocytes and the skin of C57BL/6 J mice after UVB irradiation. J Mol Med. 2023;101(5):581‐593. doi:10.1007/s00109-023-02301-y PMC10163137 37032347

[54] Lynn Lamoreux M . The inbred mouse in pigmentation research: significance of a congenic developmental system. Pigment Cell Res. 2000;13(6):421‐430. doi:10.1034/j.1600-0749.2000.130603.x 11153693

[55] You YJ , Wu PY , Liu YJ , et al. Sesamol inhibited ultraviolet radiation‐induced hyperpigmentation and damage in C57BL/6 mouse skin. Antioxidants. 2019;8(7):207. doi:10.3390/antiox8070207 PMC6680965 31284438

[56] Li H , Fan L , Zhu S , et al. Epilation induces hair and skin pigmentation through an EDN3/EDNRB‐dependent regenerative response of melanocyte stem cells. Sci Rep. 2017;7(1):7272. doi:10.1038/s41598-017-07683-x PMC5544680 28779103

[57] Nakano S , Abe Y , Nakajima K , et al. Establishment of a mouse model for post‐inflammatory hyperpigmentation. Pigment Cell Melanoma Res. 2021;34(1):101‐110. doi:10.1111/pcmr.12911 32623834

[58] Ferreira AM , de Souza AA , Koga R d CR , et al. Anti‐melanogenic potential of natural and synthetic substances: application in zebrafish model. Molecules. 2023;28(3):1053. doi:10.3390/molecules28031053 PMC9920495 36770722

[59] Neuffer SJ , Cooper CD . Zebrafish syndromic albinism models as tools for understanding and treating pigment cell disease in humans. Cancers (Basel). 2022;14(7):1752. doi:10.3390/cancers14071752 PMC8997128 35406524

[60] Liu L , Zhong M , Dong J , Chen M , Shang J , Yue Y . 5‐hydroxytryptamine (5‐HT) positively regulates pigmentation via inducing melanoblast specification and melanin synthesis in zebrafish embryos. Biomolecules. 2020;10(9):1‐12. doi:10.3390/biom10091344 PMC7563192 32961761

[61] Wusiman Z , Zhang AM , Zhang SS , et al. Galangin ameliorates PTU‐induced vitiligo in zebrafish and B16F10 cells by increasing melanogenesis through activation of the p38/JNK MAPK pathway. Front Pharmacol. 2025;16:1521097. doi:10.3389/fphar.2025.1521097 PMC11931063 40129935

[62] Kawasaki‐Nishihara A , Nishihara D , Nakamura H , Yamamoto H . ET3/Ednrb2 signaling is critically involved in regulating melanophore migration in Xenopus. Dev Dyn. 2011;240(6):1454‐1466. doi:10.1002/dvdy.22649 21538684

[63] Yi H , Liang W , Yang S , et al. Melanin deposition and key molecular features in Xenopus tropicalis oocytes. BMC Biol. 2025;23(1):62. doi:10.1186/s12915-025-02168-0 PMC11866844 40016733

[64] Bertolesi GE , Debnath N , Malik HR , Man LLH , McFarlane S . Type II opsins in the eye, the pineal complex and the skin of Xenopus laevis: using changes in skin pigmentation as a readout of visual and circadian activity. Front Neuroanat. 2022;15:784478. doi:10.3389/fnana.2021.784478 PMC8814574 35126061

[65] El Mir J , Nasrallah A , Thézé N , et al. Xenopus as a model system for studying pigmentation and pigmentary disorders. Pigment Cell Melanoma Res. 2025;38(1):13178. doi:10.1111/pcmr.13178 PMC11681847 38849973

[66] Bastide H , Yassin A , Johanning EJ , Pool JE . Pigmentation in Drosophila melanogaster reaches its maximum in Ethiopia and correlates most strongly with ultra‐violet radiation in sub‐Saharan Africa. BMC Evol Biol. 2014;14(1):179. doi:10.1186/s12862-014-0179-y PMC4236528 25115161

[67] Bolognia JL , Murrn MS , Pawelek JM . Hairless pigmented Guinea pigs: a new model for the study of mammalian pigmentation. Pigment Cell Res. 1990;3(3):150‐156. 10.1111/j.1600-0749.1990.tb00280.x 2290786

[68] Tadokoro R , Murai H , Sakai KI , Okui T , Yokota Y , Takahashi Y . Melanosome transfer to keratinocyte in the chicken embryonic skin is mediated by vesicle release associated with rho‐regulated membrane blebbing. Sci Rep. 2016;6:38277. doi:10.1038/srep38277 PMC5133614 27910904

[69] Wang Y , Herringshaw E , Anderson RR , Tam J . The Yucatan miniature swine as a model for post‐inflammatory hyperpigmentation. Pigment Cell Melanoma Res. 2024;37(3):403‐410. doi:10.1111/pcmr.13162 38361478

[70] Kiani AK , Pheby D , Henehan G , et al. Ethical considerations regarding animal experimentation. J Prev Med Hyg. 2022;63(2):E255‐E266. doi:10.15167/2421-4248/jpmh2022.63.2S3.2768 PMC9710398 36479489

[71] Lanigan TM , Kopera HC , Saunders TL . Principles of genetic engineering. Genes (Basel). 2020;11(3):291. doi:10.3390/genes11030291 PMC7140808 32164255

[72] Coleman WB . Understanding human disease in the post‐genomic era. In: Essential Concepts in Molecular Pathology. Elsevier; 2020:101‐111. doi:10.1016/B978-0-12-813257-9.00006-1

[73] Aljabali AAA , El‐Tanani M , Tambuwala MM . Principles of CRISPR‐Cas9 technology: advancements in genome editing and emerging trends in drug delivery. J Drug Delivery Sci Technol. 2024;92:105338. doi:10.1016/j.jddst.2024.105338

[74] Bhardwaj A , Nain V . TALENs: an indispensable tool in the era of CRISPR: a mini review. J Genet Eng Biotechnol. 2021;19(1):125. doi:10.1186/s43141-021-00225-z PMC8380213 34420096

[75] Yang Y , Liu Y , Wang Y , et al. Regulation of SIRT1 and its roles in inflammation. Front Immunol. 2022;13:831168. doi:10.3389/fimmu.2022.831168 PMC8962665 35359990

[76] Jeon S , Kim MM . The down‐regulation of melanogenesis via MITF and FOXO1 signaling pathways in SIRT1 knockout cells using CRISPR/Cas9 system. J Biotechnol. 2021;342:114‐127. doi:10.1016/j.jbiotec.2021.10.005 34757047

[77] Siddiqui MF , Kim MM . SIRT7 gene knockout using CRISPR/Cas9 system enhances melanin production in the melanoma cells. Biochim Biophys Acta. 2021;1867(11):166219. doi:10.1016/j.bbadis.2021.166219 34303808

[78] Song Y , Zhang Y , Chen M , et al. Functional validation of the albinism‐associated tyrosinase T373K SNP by CRISPR/Cas9‐mediated homology‐directed repair (HDR) in rabbits. EBioMedicine. 2018;36:517‐525. doi:10.1016/j.ebiom.2018.09.041 PMC6197749 30274819

[79] Karimi MA , Paryan M , Fard GB , Sadeghian H , Zarrinfar H , Bafghi MH . Challenges and opportunities in the application of CRISPR‐Cas9: a review on genomic editing and therapeutic potentials. Med Princ Pract. 2025;35:1‐17. doi:10.1159/000547334 PMC12503735 40675140

[80] Uddin F , Rudin CM , Sen T . CRISPR gene therapy: applications, limitations, and implications for the future. Front Oncol. 2020;10:1387. doi:10.3389/fonc.2020.01387 PMC7427626 32850447

[81] Li M , Zhou S , Zhang S , et al. Transdermal delivery of CRISPR/Cas9‐mediated melanoma gene therapy via polyamines‐modified thermosensitive hydrogels. J Nanobiotechnol. 2025;23(1):441. doi:10.1186/s12951-025-03523-7 PMC12164105 40514654

[82] Jayarajan V , Kounatidou E , Qasim W , Di WL . Ex vivo gene modification therapy for genetic skin diseases—recent advances in gene modification technologies and delivery. Exp Dermatol. 2021;30(7):887‐896. doi:10.1111/exd.14314 PMC8432139 33657662

[83] Takashima S , Shinkuma S , Fujita Y , et al. Efficient gene reframing therapy for recessive dystrophic epidermolysis bullosa with CRISPR/Cas9. J Investig Dermatol. 2019;139(8):1711‐1721.e4. doi:10.1016/j.jid.2019.02.015 30831133

[84] Chaudhary K , Pratap D , Sharma PK . Transcription activator‐like effector nucleases (TALENs): An efficient tool for plant genome editing. Eng Life Sci. 2016;16(4):330‐337. doi:10.1002/elsc.201500126

[85] Scott JNF , Kupinski AP , Boyes J . Targeted genome regulation and modification using transcription activator‐like effectors. FEBS J. 2014;281(20):4583‐4597. doi:10.1111/febs.12973 25124066

[86] Bedell VM , Wang Y , Campbell JM , et al. In vivo genome editing using a high‐efficiency TALEN system. Nature. 2012;491(7422):114‐118. doi:10.1038/nature11537 PMC3491146 23000899

[87] Markina NM , Kotlobay AA , Tsarkova AS . Heterologous metabolic pathways: strategies for optimal expression in eukaryotic hosts. Acta Naturae. 2020;12(2):28‐39. doi:10.32607/actanaturae.10966 PMC7385092 32742725

[88] Tran‐Ly AN , Reyes C , Schwarze FWMR , Ribera J . Microbial production of melanin and its various applications. World J Microbiol Biotechnol. 2020;36(11):170. doi:10.1007/s11274-020-02941-z PMC7548279 33043393

[89] Martínez LM , Martinez A , Gosset G . Production of melanins with recombinant microorganisms. Front Bioeng Biotechnol. 2019;7:285. doi:10.3389/fbioe.2019.00285 PMC6821874 31709247

[90] Bolognese F , Scanferla C , Caruso E , Orlandi VT . Bacterial melanin production by heterologous expression of 4‐hydroxyphenylpyruvate dioxygenase from Pseudomonas aeruginosa. Int J Biol Macromol. 2019;133:1072‐1080. doi:10.1016/j.ijbiomac.2019.04.061 31029629

[91] Zhang Q , Yang X , Lin L , et al. Pyomelanin production via heterologous expression of 4‐hydroxyphenylpyruvate dioxygenase (HPPD) and construction of HPPD inhibitor screening model. J Biosci Bioeng. 2023;135(2):93‐101. doi:10.1016/j.jbiosc.2022.10.005 36470730

[92] Ahn SY , Jang S , Sudheer PDVN , Choi KY . Microbial production of melanin pigments from caffeic acid and l‐tyrosine using streptomyces glaucescens and fcs‐ech‐expressing escherichia coli. Int J Mol Sci. 2021;22(5):1‐15. doi:10.3390/ijms22052413 PMC7957706 33673727

[93] Campana R , Fanelli F , Sisti M . Role of melanin in the black yeast fungi Aureobasidium pullulans and Zalaria obscura in promoting tolerance to environmental stresses and to antimicrobial compounds. Fungal Biol. 2022;126(11–12):817‐825. doi:10.1016/j.funbio.2022.11.002 36517149

[94] de León LR , Moreno‐Perlín T , Castillo‐Marenco T , et al. Polyextremotolerant, opportunistic, and melanin‐driven resilient black yeast Exophiala dermatitidis in environmental and clinical contexts. Sci Rep. 2025;15(1):6472. doi:10.1038/s41598-025-88595-z PMC11846982 39987208

[95] Morris‐Jones R , Gomez BL , Diez S , et al. Synthesis of melanin pigment by Candida albicans in vitro and during infection. Infect Immun. 2005;73(9):6147‐6150. doi:10.1128/IAI.73.9.6147-6150.2005 PMC1231073 16113337

[96] Walker CA , Gómez BL , Mora‐Montes HM , et al. Melanin externalization in candida albicans depends on cell wall chitin structures. Eukaryot Cell. 2010;9(9):1329‐1342. doi:10.1128/EC.00051-10 PMC2937336 20543065

[97] Ben Tahar I , Kus‐Liśkiewicz M , Lara Y , Javaux E , Fickers P . Characterization of a nontoxic pyomelanin pigment produced by the yeast Yarrowia lipolytica. Biotechnol Prog. 2020;36(2):e2912. doi:10.1002/btpr.2912 31525285

[98] d'Ischia M , Wakamatsu K , Cicoira F , et al. Melanins and melanogenesis: from pigment cells to human health and technological applications. Pigment Cell Melanoma Res. 2015;28(5):520‐544. doi:10.1111/pcmr.12393 26176788

[99] Solano F . Melanins: skin pigments and much more—types, structural models, biological functions, and formation routes. New J Sci. 2014;2014:1‐28. doi:10.1155/2014/498276

[100] Mostert AB , Powell BJ , Pratt FL , et al. Role of semiconductivity and ion transport in the electrical conduction of melanin. Appl Phys Sci. 2012;109(23):8943‐8947. doi:10.1073/pnas.1119948109 PMC3384144 22615355

[101] Chung S , Lim GJ , Lee JY . Quantitative analysis of melanin content in a three‐dimensional melanoma cell culture. Sci Rep. 2019;9(1):780. doi:10.1038/s41598-018-37055-y PMC6349835 30692593

[102] Ponmozhi J , Dhinakaran S , Varga‐medveczky Z , et al. Development of skin‐on‐a‐chip platforms for different utilizations: factors to be considered. Micromachines. 2021;12(3):1‐25. doi:10.3390/mi12030294 PMC8001759 33802208

[103] Mohamadali M , Ghiaseddin A , Irani S , Amirkhani MA , Dahmardehei M . Design and evaluation of a skin‐on‐a‐chip pumpless microfluidic device. Sci Rep. 2023;13(1):8861. doi:10.1038/s41598-023-34796-3 PMC10232512 37258538

[104] Alépée N , Grandidier MH , Cotovio J . Usefulness of the EpiSkin™ reconstructed human epidermis model within integrated approaches on testing and assessment (IATA) for skin corrosion and irritation. Toxicol In Vitro. 2019;54:147‐167. doi:10.1016/j.tiv.2018.09.015 30266437

[105] Pellevoisin C , Videau C , Briotet D , et al. SkinEthic™ RHE for in vitro evaluation of skin irritation of medical device extracts. Toxicol in Vitro. 2018;50:418‐425. doi:10.1016/j.tiv.2018.01.008 29339149

[106] Askari M , Afzali Naniz M , Kouhi M , Saberi A , Zolfagharian A , Bodaghi M . Recent progress in extrusion 3D bioprinting of hydrogel biomaterials for tissue regeneration: a comprehensive review with focus on advanced fabrication techniques. Biomater Sci. 2021;9(3):535‐573. doi:10.1039/d0bm00973c 33185203

[107] Zoller N , Hofmann M , Butting M , et al. Assessment of melanogenesis in a pigmented human tissue‐cultured skin equivalent. Indian J Dermatol. 2019;64(2):85‐89. doi:10.4103/ijd.ijd_410_17 PMC6440179 30983601

[108] Coutant K , Magne B , Ferland K , et al. Melanocytes in regenerative medicine applications and disease modeling. J Transl Med. 2024;22(1):336. doi:10.1186/s12967-024-05113-x PMC11003097 38589876

[109] Bellei B , Papaccio F , Picardo M . Regenerative medicine‐based treatment for vitiligo: An overview. Biomedicine. 2022;10(11):2744. doi:10.3390/biomedicines10112744 PMC9687162 36359263

[110] Birlea SA , Costin GE , Roop DR , Norris DA . Trends in regenerative medicine: Repigmentation in vitiligo through melanocyte stem cell mobilization. Med Res Rev. 2017;37(4):907‐935. doi:10.1002/med.21426 PMC5466503 28029168

[111] Howell RC , Schweitzer AD , Casadevall A , Dadachova EA . Chemosorption of radiometals of interest to nuclear medicine by synthetic melanins. Nucl Med Biol. 2008;35(3):353‐357. doi:10.1016/j.nucmedbio.2007.12.006 PMC2516407 18355691

[112] Shi G , Feng Y , Tonissen KF . Development of a human tyrosinase activity inhibition assay using human melanoma cell lysate. Biotechniques. 2024;76(11):547‐551. doi:10.1080/07366205.2024.2441637 39679544

[113] Lv J , Meng D , Zhang H , et al. Apigenin promotes melanogenesis and melanosome transport through the c‐KIT/Raf‐1/MAPK/CREB pathway in HEMCs. Front Pharmacol. 2025;16:1572878. doi:10.3389/fphar.2025.1572878 PMC12066314 40356959

[114] Yang YM , Son YO , Lee SA , Jeon YM , Lee JC . Quercetin inhibits α‐MSH‐stimulated melanogenesis in B16F10 melanoma cells. Phytother Res. 2011;25(8):1166‐1173. doi:10.1002/ptr.3417 21290442

[115] Yao C , Oh JH , Oh IG , Park CH , Chung JH . [6]‐Shogaol inhibits melanogenesis in B16 mouse melanoma cells through activation of the ERK pathway. Acta Pharmacol Sin. 2013;34(2):289‐294. doi:10.1038/aps.2012.134 PMC4011614 23123645

[116] Jafarzadeh A , Hoseini SS , Behrangi E , Roohaninasab M , Goodarzi A . Effectiveness of regenerative medicine for skin lightening and rejuvenation: a systematic review of extracellular vesicles and conditioned media. Stem Cell Res Ther. 2025;16(1):513. doi:10.1186/s13287-025-04592-z PMC12465346 41013717

[117] Zaaijer S , Groen SC . Implementing differentially pigmented skin models for predicting drug response variability across human ancestries. Hum Genomics. 2024;18(1):113. doi:10.1186/s40246-024-00677-7 PMC11465898 39385300

[118] Cubillos‐Ruiz A , Guo T , Sokolovska A , et al. Engineering living therapeutics with synthetic biology. Nat Rev Drug Discov. 2021;20(12):941‐960. doi:10.1038/s41573-021-00285-3 34616030

[119] Vasudevan S , Vogt WC , Weininger S , Pfefer TJ . Melanometry for objective evaluation of skin pigmentation in pulse oximetry studies. Commun Med. 2024;4(1):138. doi:10.1038/s43856-024-00550-7 PMC11239860 38992188

